# Wild *Rosa* Endophyte M7SB41-Mediated Host Plant’s Powdery Mildew Resistance

**DOI:** 10.3390/jof9060620

**Published:** 2023-05-27

**Authors:** Yi Zhao, Wenqin Mao, Wenting Tang, Marcos Antônio Soares, Haiyan Li

**Affiliations:** 1 Key Laboratory of Chemistry in Ethnic Medicinal Resources, Yunnan Minzu University, Kunming 650500, China; febflower-zy@163.com; 2 Life Science and Technology & Medical Faculty, Kunming University of Science and Technology, Kunming 650500, China; wenqinmao@icloud.com (W.M.); wentwtxz@163.com (W.T.); 3 Department of Botany and Ecology, Federal University of Mato Grosso, Cuiabá 78060-900, Brazil; drmasoares@gmail.com

**Keywords:** powdery mildew, *Seimatosporium* sp., resistance mechanism, transcriptomics, salicylic acid

## Abstract

Our previous studies indicated that endophyte M7SB41 (*Seimatosporium* sp.) can significantly enhance host plants powdery mildew (PM) resistance. To recover the mechanisms, differentially expressed genes (DEGs) were compared between E+ (endophte-inoculated) and E− (endophyte-free) plants by transcriptomics. A total of 4094, 1200 and 2319 DEGs between E+ and E− were identified at 0, 24, and 72 h after plants had been infected with PM pathogen *Golovinomyces cichoracearum*, respectively. Gene expression pattern analysis displayed a considerable difference and temporality in response to PM stress between the two groups. Transcriptional profiling analysis revealed that M7SB41 induced plant resistance to PM through Ca^2+^ signaling, salicylic acid (SA) signaling, and the phenylpropanoid biosynthesis pathway. In particular, we investigated the role and the timing of the SA and jasmonic acid (JA)-regulated defensive pathways. Both transcriptomes and pot experiments showed that SA-signaling may play a prominent role in PM resistance conferred by M7SB41. Additionally, the colonization of M7SB41 could effectively increase the activities and the expression of defense-related enzymes under PM pathogen stress. Meanwhile, our study revealed reliable candidate genes from TGA (TGACG motif-binding factor), WRKY, and pathogenesis-related genes related to M7SB41-mediate resistance. These findings offer a novel insight into the mechanisms of endophytes in activating plant defense responses.

## 1. Introduction

Powdery mildew (PM) is one of the most common and important fungal diseases of plants worldwide [1]. It is caused by obligate biotrophic fungi of the order Erysiphales which attack nearly 10,000 species of angiosperms, including both dicot and monocot plants [2,3,4]. Even though the pathogen rarely kills the plant directly, heavy infections may reduce host plant vigor, productivity, market value, and consumer acceptance. Until now, in practice, the application of fungicides has been the primary strategy for managing PM [4]. However, it often leads to the emergence of high-risk PM strains resistant to several fungicide classes. Therefore, it is crucial to develop more suitable and promising alternative control strategies [5]. Along with selection of resistant plants, biological control by beneficial microorganisms is increasingly considered as one of the most promising technologies for its sustainability, environment-friendliness, and cost-effectiveness [6,7].

Endophytes are microorganisms that reside asymptomatically in the interior of host plant tissues [8]. Compared to other biocontrol agents, the application of endophytes in biological control provides potential advantages since they reside within host plants and thereby are protected from environmental stresses [9]. As extremely important plant partners, endophytes have a profound influence on the physiological and pathological functions of plants [10,11]. Some endophytic fungi (EF) have been proved to modify disease severity by interacting directly with pathogenic competitors (e.g., competition and mycoparisitism) and/or by improving the natural resistance of a plant [12,13]. They can also afford host cross-protection against plant pathogenic fungi by producing a number of antibiotics, toxins, and biologically active secondary metabolites [14]. Further evidence shows that colonization with some endophytes can activate not only defensive enzymes or genes in plants, but also induce the synthesis of phytohormones, which thus results in acquisition of induced resistance [15].

Systemic acquired resistance (SAR) and induced systemic resistance (ISR) are two main pathways in the plant immune system [16]. In most cases, SAR depends on salicylic acid (SA) signaling, whereas ISR is regulated by jasmonic acid (JA) and ethylene (ET) signaling [17,18]. Signaling crosstalk between SA, JA/ET mediated pathways allows the host to finely adjust the defense response. Beneficial endophytes may trigger one or both JA/ET or SA-dependent signalings in different pathosystems [19,20]. Currently, it is still unclear about the complex hormone signaling underlying the induction of resistance.

*Rosa multiflora* Thunb is a native wild *Rosa* cultivar of Yunnan Province in China, which is resistant to PM [21]. *Rosa multiflora* var. *carnea* Redouté and Thory is a variant of *R*. *multiflora*. However, it is highly susceptible to PM [21,22]. Our previous studies have revealed that there were significant differences between susceptible and tolerant varieties in terms of diversity and community structure of EF [23]. It suggests that endophytes may play a role in enhancing the resilient property of the resistant cultivars.

M7SB41 is a culturable endophyte that was isolated from *R. multiflora* Thunb, and identified to be *Seimatosporium* sp. Our pot experiments have demonstrated that M7SB41 effectively reduced the severity of tobacco PM [24]. However, the mechanisms employed by M7SB41 in PM control are still unclear. The objective of this study was to elucidate how M7SB41 enhanced host plants PM resistance. Comprehensive transcriptome analysis can provide an ideal platform to investigate the genome-wide differentiations underlying the differently physiological traits, as well as to uncover complex signaling pathways activated in response to pathogen infection [25]. Therefore, to elucidate the mechanisms involved in host plants PM resistance induced by M7SB41, the differentially expressed genes between E+ and E− plants were studied by transcriptomic techniques. Moreover, the activity of β-1, 3-glucanase, and the production of phytohormones SA and JA between E+ and E− plants infected with PM were also compared.

## 2. Materials and Methods

### 2.1. Isolation of M7SB41 and Source of Powdery Mildew Pathogen

The strain M7SB41 was isolated from surface-sterilized stems of the known PM resistant rose cultivar *R. multiflora* Thunb, which was collected from Cangshan Mountain, Yunnan Province, Southwest China (25°25′–27°58′ N, 99°58′–100°27′ E) [21,23]. The isolate was identified as *Seimatosporium* sp. based on molecular analysis and morphological characteristics (Genbank accession number: MH051045). Strain M7SB41 was stored in the microbiological laboratory of the Medical School, Kunming University of Science and Technology. Powdery mildew pathogen *Golovinomyces cichoracearum*, (DC.) V.P. Heluta (ex *Erysiphe cichoracearum* DC.) was provided and identified by Xie [26]. For plant infection, fresh PM pathogen spores were brushed from natural infected tobacco (*Nicotiana tabacum* K326) leaves showing typical symptoms of PM and put in 100 mL of sterile distilled water containing 2 drops of Tween-80 for spore suspension. The suspension was centrifuged at 1500 rpm for 10 min twice, then the conidia were final adjusted to 1 × 10^6^ conidia/mL.

### 2.2. Plant Culture and Inoculation with Endophyte M7SB41 and the Powdery Mildew Pathogen

Seeds of *N. tabacum* K326 were provided by Yuxi China Tobacco Seed Co., Ltd. (Yuxi, China). These seeds were surface-sterilized as follows: seeds were immersed in 75% ethanol for 2 min, then in 5% sodium hypochlorite solution for 2 min, and rinsed three times with sterile distilled water. Subsequently, they were dried on sterile filter paper [27]. These seeds were germinated in trays filled with sterilized soil substrate (70% peat moss, 30% perlite, *v*/*v*, Fafard, Saint-Bonaventure, QC, Canada) for 60 days in a growth chamber (16/8 h light/dark cycle, 26/18 °C, relative humidity of 60%). Seedlings were regularly watered with deionized-distilled water three times weekly, and supplied with Hoagland’s nutrient solutions once a week. After 60 days, the healthy plants with similar size were selected and transferred to plastic pots (one seedling per pot). 

To prepare the inoculum, strain M7SB41 was cultured in PDB medium (200 g potato, 20 g glucose, 1000 mL distilled water) at 25 °C for 4–6 days at 125 rpm. The mycelia were collected, then cut into as small pieces as possible using sterilized ophthalmic scissors and diluted with sterilized water. The endophyte-infected plants (E+) were sprayed with M7SB41 inoculums twice over 14 days (at 7, 14 days after transplanting). While the endophyte-free plants (E−) were sprayed with the autoclaved inoculums of M7SB41 (121 °C, 15 psi for 15 min to kill the fungi) in equal amount. Seven days after the endophyte inoculation, the surface of each seedling (E+ and E−) was sprayed with fresh pathogen spores (1 × 10^6^ conidia /mL). The plants were randomly placed and kept under controlled conditions at 25 *±* 2 °C, 16-h light, and 8-h dark.

For SA, JA analysis, the leaves of E+ and E− were collected at 0 d (before PM pathogen infection), 7 and 10 d (after PM pathogen infection) of the spray, respectively. There were three replicate pots for each treatment at each time-point. As for β-1, 3-glucanase, enzyme activities were only determined in the 7 d samples (ten biological replicates of each treatment). For RNA sequencing, fresh leaves were collected at time point 0 h (before PM pathogen infection), 24 and 72 h post-infection. Fresh leaf samples were frozen in liquid nitrogen and stored at −80 °C. 

### 2.3. β-1,3-Glucanase Activity Analysis

Leaves (about 100 mg) were crushed in liquid nitrogen and then centrifuged at 12,000× *g* for 10 min at 4 °C. The supernatant was assayed for β-1, 3-glucanase activity. Commercial glucose oxidase kits (Solarbio, Beijing, China) were used for the determination. One unit of β-1, 3-glucanase activity was defined as the quantity of enzyme required for liberating 1 mg of glucose equivalent per gram per hour of fresh plant samples.

### 2.4. SA and JA Contents Assay

SA was rapidly extracted based on previously described methods with modifications [28,29]. Two g of frozen leaves were pulverized. Then, 20 mL cold 50% methanol and 30 mL cold petroleum ether were added for extraction. The extracts were dissolved in 2.0 mL methanol and filtered through a 0.22 mm filter prior to ESI-UHPLC-MS/MS analysis. Analysis of SA was performed using a Vanquish UPLC system equipped with a TSQ QUANTIS Mass Spectrometer (Thermo Fisher, Waltham, MA, USA). An amount of 2 × 10^−3^ mL crude extraction of SA was injected into a Poroshell 120 SB-C18 reverse-phase column (diameter × length: 2.1 × 150 mm, 2.7 μm particle size, Agilent) at 30 °C. The mobile phase consisting of CH_3_OH with 0.1% CH_2_O_2_ (phase A) and H_2_O with 0.1% CH_2_O_2_ (phase B). SA was annotated and quantified in chromatograms of authentic standard mixtures by its retention times. Characteristic MS/MS fragmentation patterns and *m*/*z* values of corresponding [M– H]^−^ ions are summarized in Table S1.

Extraction of JA was performed as follows: 0.5 g frozen leaf tissues were stirred in 1.2 mL phosphate buffered solution (pH 7.4), and further homogenized at 2500 rmp at 4 °C for 40 min. Supernatants were assayed according to the plant jasmonic acid ELISA Kit (mlbio, Shanghai, China).

### 2.5. RNA Extraction and Illumina Sequencing

Frozen leaves (0.1 g) were used for total RNA extraction using Omni Plant RNA kit (Cwbio, Taizhou, China) following the manufacturer’s recommendations. The integrity and purity of RNA was determined by agarose gel electrophoresis and the concentration was measured using an Agilent Bioanalyzer 2100.

A cDNA library was constructed for the 18 mixed RNA samples. There were three biological replicates at each time point (0, 24, and 72 h). The resulting cDNA libraries were then paired-end sequenced (2 × 150 bp) using Illumina HiSeq 2500 (Illumina, San Diego, CA, USA). To get high quality clean reads, we performed adapter trimming and quality filtering with fastp (version 0.18.0) [30]. Briefly, adapter contaminations were removed from the raw reads. Then low-quality reads (>50% bases with quality scores Q-value ≤ 20, and/or Ns > 10%) were removed from raw data to obtain more reliable results. Clean reads were then aligned against the tobacco reference transcriptome by TopHat2 (Version 2.0.3.12) and analyzed using the Cufflinks package [31]. Gene expression levels were normalized based on fragments per kilobase of exon per million mapped read (FPKM) values.

To predict the transcriptional changes over time in the two groups (E+ and E−) under PM stress, expression of differentially expressed genes (DEGs) at the same time points were analyzed (C0 vs. T0, C24 vs. T24, C72 vs. T72). Here, DEGs are those genes that showed significantly (fold change ≥ 2 and FDR adjusted *p*-value < 0.05) different transcript levels among comparisons, and the significance of the differences in gene expression was measured by the edgeR package [32]. Assignment and annotation of functional categories for DEGs were based on the enrichment theory of gene ontology (GO) classification and the Kyoto Encyclopedia of Genes and Genomes (KEGG) pathways. The threshold of *p* < 0.05 was selected as identifying significantly.

To assess the dynamic transcription profiles of each group, expression pattern analysis was performed using Short Time-Series Expression Miner (STEM) version 1.3.11. The Illumina sequencing data obtained in these experiments are publicly available in the NCBI Sequence Read Archive under No. PRJNA649603.

### 2.6. Validation by qRT-PCR

A total of 8 genes significantly differentially expressed transcripts were randomly selected for validation using quantitative real-time PCR (qRT-PCR) analysis. Specific primers were designed by Primer Premier 5.0 (Table S2). Leaves were collected as in the above methods. Total RNA was then extracted from E+ and E− plants using the Plant RNA rapid extraction kit (Zoonbio Biotechnology, Nanjing, China). First strand complementary DNA was synthesized by the Prime Script Ⅱ 1st Strand cDNA Synthesis Kit (TaKaRa, Shiga, Japan), followed by quantitative PCR using SYBR Premix Ex Taq (TaKaRa, Shiga, Japan). Elongation factor 1α (Ef1α) was used as an internal reference [33]. The conditions for amplification were as follows: 1 cycle at 95 °C for 30 s, followed by 40 cycles of 95 °C for 5 s, 60 °C for 20 s, and 72 °C for 20 s. Melting curves were determined as follows: 95 °C for 0 s, 65 °C for 15 s, and 97 °C for 0 s on the continuous acquisition mode. All qPCR reactions were carried out in three technical replicates for each gene. The relative expressions were calculated by the 2^−ΔΔCt^ method [34].

### 2.7. Statistical Analysis

The statistical analyses were performed with SPSS 19.0 (IBM, Armonk, NY, USA). Student’s *t*-test was performed to detect differences in β-1, 3-glucanase activity between E+ and E− plants. The contents of SA and JA were analyzed by one-way ANOVA. When ANOVA assumptions were not met, the Kruskal–Wallis test in SPSS was performed to determine the differences among the different treatments [35].

## 3. Results

### 3.1. Activity of β-1, 3-Glucanase

**Figure 1:**
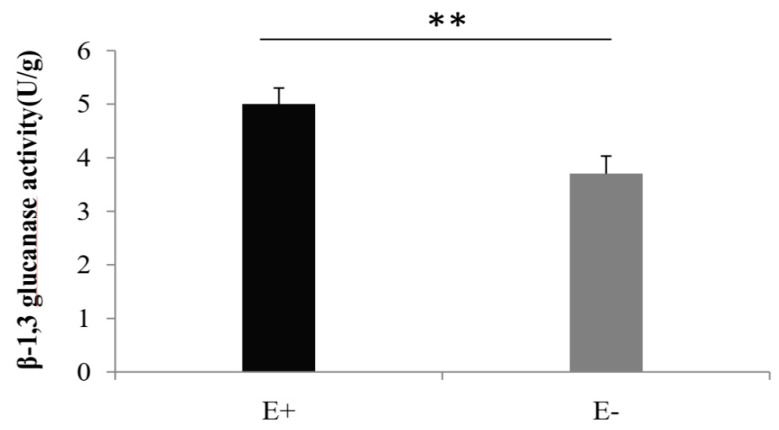
β-1,3-glucanase activity in tobacco leaves after interaction with M7SB41 under pathogen stress. E+ and E− means endophyte-infected and endophyte-free plants, respectively. Values expressed as the means ± SD (n = 10), Student’s *t*-test was used to determine the difference between E+ and E− plants. Variation between E+ and E− were indicated by ** *p* < 0.01.

To quantify the elicited defense response on a biochemical level, β-1,3-glucanase activity was determined in buffered tobacco extracts at 7 d (after PM pathogen infection) (Figure 1). The results showed that β-1,3-glucanase activities of E+ were significantly increased, suggesting that M7SB41 may affect the host plants PM resistance by regulating defense relate enzyme activity (*p* < 0.01, Student’s *t*-test).

### 3.2. Concentration of SA and JA

**Figure 2:**
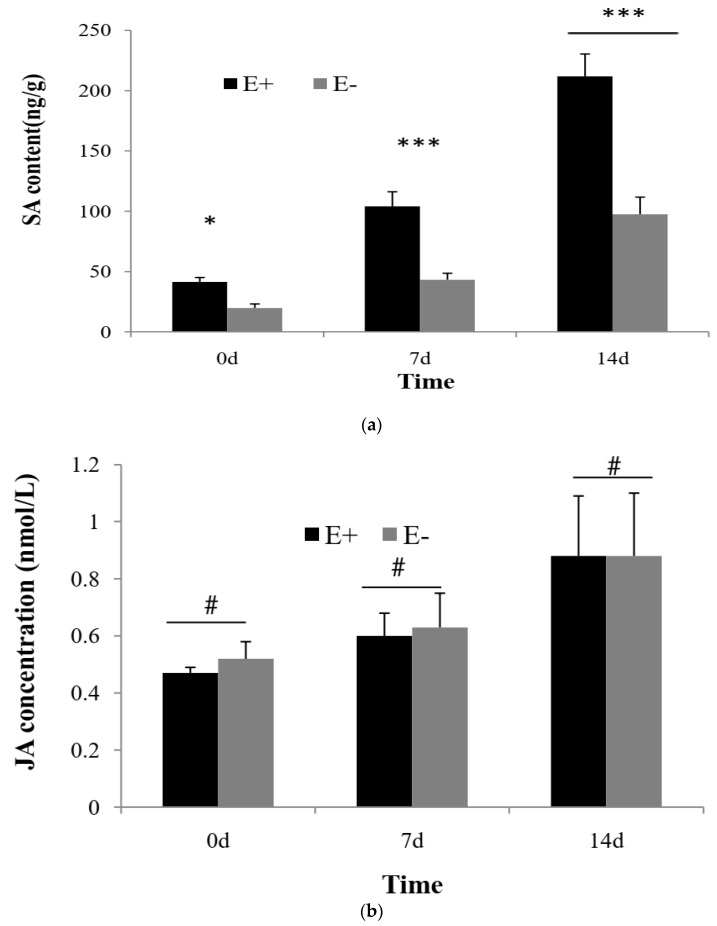
Effects of M7SB41 on the content of SA and JA in tobacco under pathogen stress. (**a**) The contents of SA at 0, 7, 14 d after challenge with *G. cichoracearum*. (**b**) The contents of JA at 0, 7, 14 d after challenge with *G. cichoracearum*. E+ and E− means endophyte-infected and endophyte-free plants, respectively. Values expressed as the means ± SD (n = 3). One-way ANOVA was performed for analysis among groups. Variations between E+ and E− were indicated by * *p* < 0.05, *** *p* < 0.001, ^#^ *p* > 0.05.

The inoculation of M7SB41 to tobacco K326 affected host plants’ SA content (Figure 2a). SA content of E+ plants was significantly higher than that of E− plants at three collecting times. (*p* < 0.05, One-way ANOVA). The concentration of SA in E+ plants was 41.33 ± 3.71, 104.04 ± 12.11, and 211.89 ± 18.49 ng/g at 0, 7, and 14 d, respectively. While the concentration of SA in E− plants was 19.82 ± 3.32, 43.19 ± 5.40, and 97.57 ± 14.12 ng/g, respectively. Moreover, SA was found to accumulate rapidly on E+ and E− plants, especially at 7 and 14 d after inoculation of *G. cichoracearum* (Figure 2a). The chromatogram and mass spectrum of SA in E+ and E− plants are shown in Figure S1.

The content of JA increased in E+ and E− plants at 7 and 14 d after inoculation of *G. cichoracearum* (Figure 2b). Moreover, JA content of E+ plants was comparatively less than that of E− plants at 0 and 7 d. However, the difference was non-significant (*p* > 0.05).

### 3.3. RNA Sequencing Yields

Transcript levels between E+ and E− plants at 0, 24, and 72 h after inoculation with PM were analyzed by Illumina sequencing. After stringent filtering, a total of 106.88 Gb clean bases reads (702,661,546 total high-quality reads) were generated from the 18 cDNA libraries Among them, 318,704,776 high-quality clean reads were detected in E+ plants and 383,956,770 high-quality clean reads were detected in E− plants. In addition, the Q30 (quality score is 30) of clean reads in each group is above 91.61% and the GC contents of all data were between 43.64% and 44.78% (Table 1). Approximately 87% to 93% of the sequenced reads were successfully aligned to the tobacco genome reference sequence using TopHat2 software (Table S3). In order to improve accuracy, the reconstruction of transcripts was carried out with software Cufflinks [36]. Finally, a total of 61,780 high-confidence transcripts were obtained across all the samples. These transcripts were used for further downstream differential expression analysis.

### 3.4. Differential Expressed Gene between E+ and E− at the Same Collecting Time

**Figure 3:**
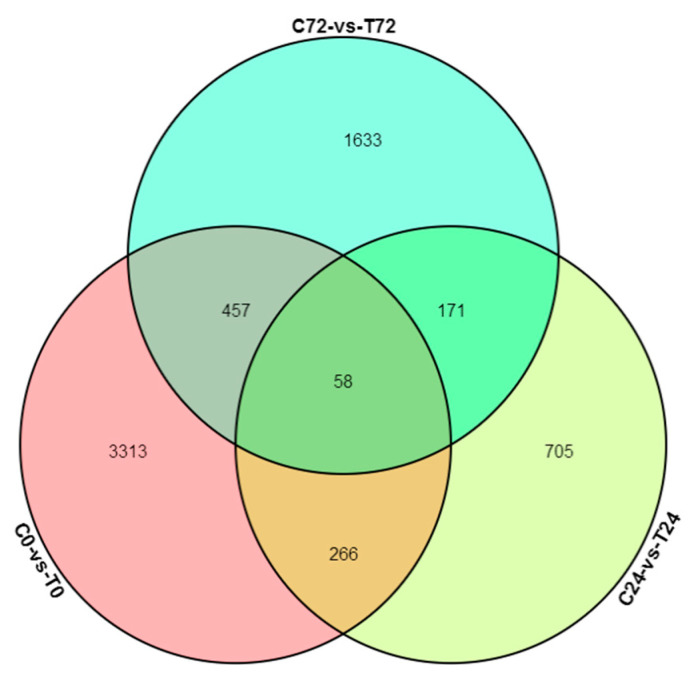
The Venn diagram of differentially expressed genes (DEGs) between E− and E+. C and T: control (E− plants) and treatment (E+ plants), respectively; 0, 24, and 72 represent 0, 24, and 72 h of infection with powdery mildew, respectively.

A total of 7613 transcripts was found to be significantly differentially expressed between E+ and E− plants. They were identified by three pairwise comparisons of transcriptome datasets (C0 vs. T0, C24 vs. T24, C72 vs. T72). Among them, 4094 DEGs (2268 up-regulated and 1826 down-regulated) were identified at 0 h, 1200 (767 up-regulated and 433 down-regulated) at 24 h, and 2319 (1045 up-regulated and 1274 down-regulated) at 72 h (Figure S2). The Venn diagram illustrated the distribution of DEGs at each collection time (Figure 3). It was found that 58 genes were differentially expressed at 0, 24, and 72 h.

Although more DEGs were observed at 0 h than that of 24 and 72 h, there were no significant differences in the numbers of GO terms and KEGG pathways for all three collecting times, i.e., 36, 34, and 35 enriched GO terms and 145, 104, and 139 enriched KEGG pathways for 0, 24, and 72 h respectively. Inoculation with M7SB 41 led to enrichment of GO terms, including metabolic process, cellular process, single-organism process, response to stimulus, catalytic activity, and binding (Figure S3). At 0 h, DEGs were induced by M7SB41 without PM pathogen infection, KEGG enrichment pathway including cysteine and methionine metabolism, DNA replication, glutathione metabolism (Figure S4a). After inoculation with M7SB41 for 24 h, the top three of the enrichment pathway were starch and sucrose metabolism, biosynthesis of secondary metabolites, plant hormone signal transduction (Figure S4b); after 72 h, including protein processing in endoplasmic reticulum, glyoxylate and dicarboxylate metabolism, carbon fixation in photosynthetic organisms (Figure S4c).

### 3.5. Gene Expression Pattern Analysis

**Figure 4:**
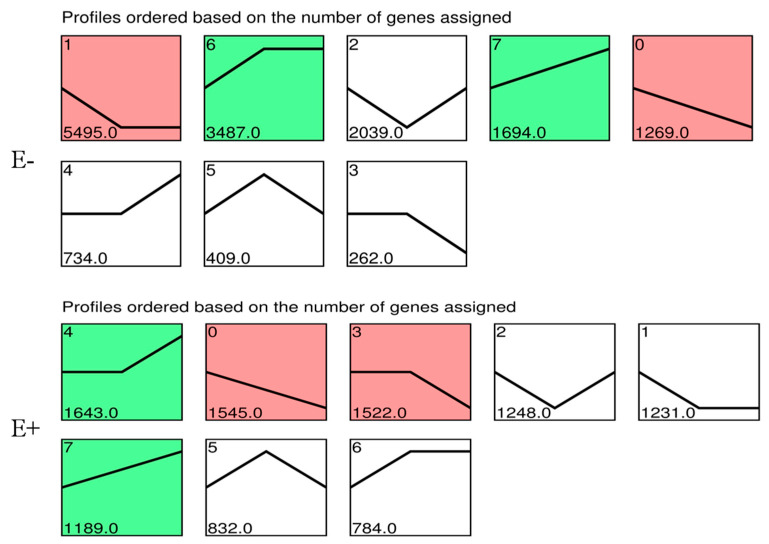
Patterns of gene expressions across three time points in the E+ plants and E−plants inferred by STEM analysis. Note: The number in top left corner indicates the profile ID number, the number in bottom left corner indicates the number of genes in that profile, and the profiles were ordered based on the number of genes. The significance is characterized by different colors (green and red represent *p*-value ≤ 0.05, red represents the most significant difference.

Based on similarity of gene expression pattern, differentially expressed genes of E+ and E− plants at three collecting times were clustered in eight distinct profiles. These profiles exhibited clear difference in gene expression over time in response to PM pathogen stress between E+ and E− plants (Figure 4). In E− plants, 5495 genes in profile 1 (*p* < 0.05) were consistently down-regulated from 0 to 24 h and reached the lowest point at 24 h. Conversely, 3487 genes in profile 6 (*p* < 0.05) were consistently up-regulated from 0 to 24 h and reached the highest point at 24 h. Then, they retained this level until 72 h. Accordingly, genes were overrepresented in the profiles with apparent changes in expression level at 24 h. In E+ plants, 1643 genes in profile 4 (*p* < 0.05) were consistently up-regulated from 24 to 72 h and reached the highest point at 72 h, while 1545 genes in profile 0 (*p* < 0.05) were consistently down-regulated from 0 to 72 h and reached the lowest point at 72 h. Therefore, the major transcriptional changes in E+ plants occurred at 72 h (profile 4, 0, and 3, *p* < 0.05) (Figure 4).

To assess the functional significance of the transcriptional changes, KEGG classifications were performed for the DEGs belonging to overrepresented profiles. In E− plants, genes involved in plant hormone signal transduction (ko04075), amino sugar and nucleotide sugar metabolism (ko00520), and biotin metabolism (ko00780), were enriched in Profile 1, where gene expressions continuously decreased from 0 to 24 h (Figure S5a). A similar pattern was observed in E+ plants; genes involved in biosynthesis of antibiotics (ko01130), secondary metabolites (ko00520), and the citrate cycle (ko00020) were enriched in Profile 1 (Figure S5b). As for Profile 4, genes involved in sesquiterpenoid and triterpenoid biosynthesis (ko00909), galactose metabolism (ko00052), glycosphingolipid biosynthesis globo and isoglobo series (ko00603) were enriched in E− plants (Figure S5c). While, in E+ plants, genes involved in valine, leucine, and isoleucine degradation (ko00280), protein processing in endoplasmic reticulum (ko04141), and starch and sucrose metabolism (ko00500) were enriched (Figure S5d).

### 3.6. Expression of Plant Defense-Related Genes

**Figure 5:**
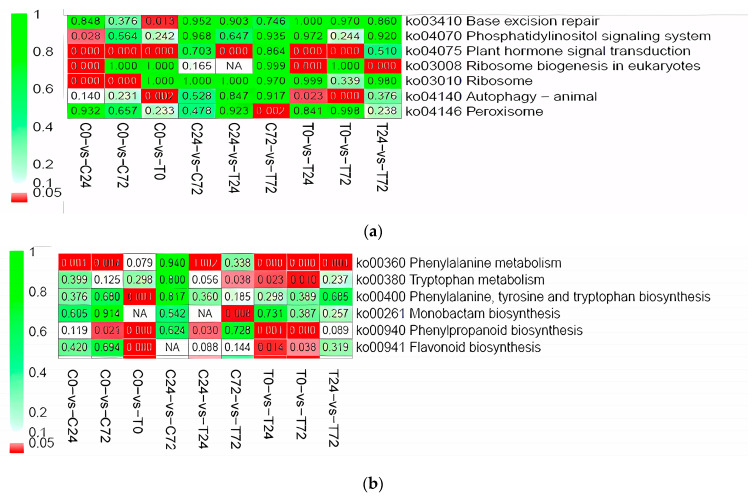
KEGG heat map of two plant defense-relate pathways. (**a**) KEGG heat map of hormone signal transduction pathway. (**b**) KEGG heat map of phenylpropanoid biosynthesis. *p* value is characterized by different colors, as shown in the example on the right, and red indicates *p* < 0.05; C and T: control (E− plants) and treatment (E+ plants), respectively; 0, 24, and 72 represent 0, 24, and 72 h of infection with powdery mildew, respectively.

Inoculation with M7SB41 induced a substantial amount of plant defense-related gene expression under PM stress. At 0 and 24 h, the KEGG-association plant hormone signal (Ko04075) of E+ was significantly increased when compared with E− (*p* < 0.05) (Figure 5a, C0 vs. T0, C24 vs. T24). There were five homologues of the TGA genes, twenty-six WRKY family genes, one MES3 gene and two JAR1-like isoform genes assigned to the ko04075 pathway (Table S4). At 0 hpi, TGA1-like, TGA1a-like, WRKY6, WRKY7, WRKY11, WRKY22-like, WRKY31, WRKY41, WRKY53, WRKY65, WRKY68, methyl salicylate esterase 3 (MES3), and jasmonate-resistant 1 (JAR1)-like were significantly high expressed in E+ plants when compared with E− plants. However, WRKY3, WRKY4, and WRKY40 were markedly low expressed at E+. At 24 h, TGA6 and WRKY7 were high expressed in E+ plants, while WRKY69 isoform and WRKY70 isoform was low expressed in E+ plants. At 72 h, high expression of WRKY41 was observed in E+ plants, while TGA6-like and WRKY50 transcripts were low expressed in E+ plants (Table S4).

Similarly, a pathway of phenylpropanoid biosynthesis (ko00940) was also enriched in E+ plants at 0 and 24 h (*p* < 0.05) (Figure 5b, C0 vs. T0, C24 vs. T24). Genes involved in the pathway were evaluated. Two key enzymes responsible for phenylpropanoid biosynthesis were upregulated in E+ plants (Table S4). The expression of 4-coumarate-CoA ligase (4CL: XM_016584785.1) was markedly increased at 0 hpi. Furthermore, two phenylalanine ammonia-lyase (PAL: XP_009759945.1; XM_016588500.1) genes were significantly induced in E+ plants both at 24 and 72 h. Other major components, important in secondary metabolism and phenylpropane biosynthesis were also differentially expressed. Among them, eleven hydroxycinnamoyl-CoA shikimate/quinate hydroxycinnamoyl transferase (HCT) genes (includes HCT-like and HCT-like isoform genes) were induced at 0 h and 72 h, ten up-regulated and one down-regulated (Table S4). Several peroxidase (POD) genes (POD3, 6, 19, 21; POD3, 7, 12, 48, 51, 63, 66, N1-like) were induced in the whole process of the infection (Table S4). 

For better understanding, the interactions among endophyte, plant, and pathogen, genes involved in plant–pathogen interaction (ko04626) pathway were noted. Genes involved in calcium/calmodulin-mediated defense signaling were found differentially expressed (Table S4). This included eleven calmodulin-like proteins (CML1, CML3, CML18, CML21, CML41, CML44), one calmodulin gene (CaM) and two CDPK genes. Interestingly, a large number of pathogenesis-related proteins (PR) were expressed differentially at 0 h (Table S4), including PR-1like, PR-5 like, PR-5 like isoform, and PR-10a like. Most of these genes were upregulated in E+ plants compared to E− plants. While, a greater number of PR genes were down-regulated at 72 h, including PR-1b, PR-1c, PR-1 like, PR-4a, PR-4b, PR-5 like isoform, and PR-10a like (Table S4).

### 3.7. RNA Sequencing Validation by qRT-PCR

**Figure 6:**
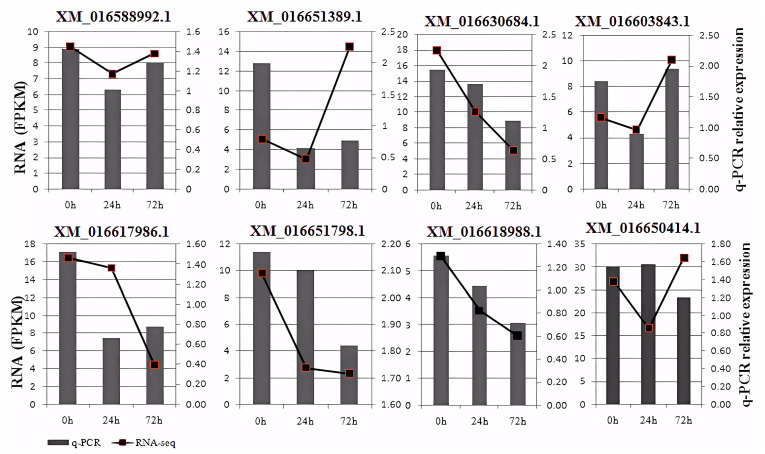
Validation of mRNA sequencing (RNA-seq) data by quantitative real-time PCR (qRT-PCR). Note: FPKM indicates fragments per kilobase of exon per million mapped reads.

qRT–PCR was used to validate eight of the DEGs identified by RNA-Seq in E+ and E− plants. Five genes, including JAR1-like isoform (XM_016588992.1, XM_016630684.1), CERK1 (XM_16603843.1), WRKY31 (XM_016651798.1),WRKY7 (XM_016618988.1) exhibited similar expression patterns at the three collecting times comparing with the transcriptome sequencing results. (Figure 6). These results indicated RNA-Seq analysis in the present study could reliably capture changes in gene expression.

## 4. Discussion

Plants have evolved a complex system to recognize microbial infection and respond adequately by activating appropriate defense responses. According to the zigzag model, there are two types of immunity to microbial pathogens, PTI (PAMP-triggered immunity) and ETI (Effector-triggered immunity) [37]. At the onset, E+ plants were pre-inoculated with M7SB41. Plants recognize the pathogen-associated molecular patterns (PAMPs) to activate PTI. During PTI, defensive enzymes such as β-1, 3-glucanases were up-regulated. Later, plants were infected by PM. The pathogen interferes with the PTI by effectors, resulting in PTI attenuate. Further, plants specifically recognize pathogen effectors by disease resistance (R) proteins to trigger ETI, which could effectively enhance resistance [38].

β-1, 3-glucanases are a well-recognized PR-2 family [39]. They play a crucial role in plant defense responses which degrade the fungal cell wall, bringing direct suppression of fungal pathogen activity and/or death [40]. β-1, 3-glucanases may be strongly induced after encountering fungal infections. In this study, β-1, 3-glucanase activities in E+ plants significantly increased at 7-day post inoculation with PM pathogen compared with E− plants. Similarly, Cao et al. [41] found that endophytes *Choiromyces aboriginum* produced higher activities of β-1, 3-glucanases which allowed them to bore holes into soilborne plant pathogens. A recent study showed that β-1, 3-glucanases safeguard the plants from fungal pathogens via oligosaccharide elicitor formation, which induces the signaling cascades for the activation of systemic and localized defense responses [42]. Therefore, these findings indicated that the change of β-1, 3-glucanase activities may be one of the mechanisms underlying endophyte M7SB41 protection of host plants against PM infection.

Different metabolites come from different gene expression, therefore, we further elucidated the regulatory networks and mechanisms of resistance to host plant PM resistance induced by M7SB41 via mRNA-sequencing (RNA-seq) techniques. The RNA-Seq results revealed several reliable candidate genes and vital pathways related to different resistance-regulating mechanisms.

### 4.1. M7SB41 Induced Changes in Ca^2+^-Mediated Defense Signaling

Ca^2+^ is a universal second messenger in all eukaryotes including plants [43]. It has been known that Ca^2+^ signals are clearly connected to activate plant immunity [44]. Accumulating evidence indicates that silencing the expression of CaM/CML gene in mutated plants strongly affects immune responses and disease resistance of plants [45,46]. Moreover, recent result revealed that VpCDPK9 and VpCDPK13 (two paralogous CDPKs from *Vitis pseudoreticulata*) contribute to grapevine PM resistance through priming SA and ET regulated defenses [47]. Here, several genes related to CaM/CML and CDPKs transcripts were up-regulated and/or down-regulated in E+ plants (Table S4). Thus, it is reasonable to assume that the colonization of M7SB41 may trigger an influx of calcium ions, then followed by changes in Ca^2+^-mediated defense signaling. This initial change is then amplified into downstream-signaling changes that may ultimately lead to major changes in defense responses.

### 4.2. Roles of WRKY TFs in Defense Responses Related to M7SB41

After Ca^2+^ signals are instantaneously activated, the expression of downstream TFs such as WRKY TFs may be regulated [48]. CDPKs may target WRKY TFs, and then affect defense hormones locally or systemically [49]. In the current study, twenty-six WRKY genes in tobacco exhibited significant differences. Among them, WRKY7 transcripts (XM_016610715.1, XM_016618988.1, XM_016619314.1, XM_016643087.1, XM_016644013.1) in E+ plants at 0 and 24 h increased compared with E− plants (Supplementary Table S4). It is known that WRKY 7, 8, 9, and 11 are required for PTI and ETI ROS burst via the MEK2 cascade [50]. Additionally, WRKY41 (XM_016638899.1) and WRKY41 isoform (XM_ 016624990.1) were overexpressed in E+ plants. WRKY41 seems to be a crucial regulator in the cross talk of SA and JA dependent defense pathways. Constitutive expression of *PR5* (SA-responsive genes) was observed in *Arabidopsis* WRKY41-overexpressing. It was also observed that overexpression of WRKY41 inhibited the expression of *PDF1.2* gene (JA-responsive genes) [48,51]. On the other hand, it was found that WRKY 40 genes (XM_016621642.1, XM_016651389.1) were down-regulated in E+ plants at 0 h (Table S4). The result was consistent with previous findings. Pandey et al. [52] found that WRKY 40 and WRKY 18 act as negative regulators of Arabidopsis PM pathogen *Golovinomyces orontii* resistance in a partially redundant manner. WRKY 40 and WRKY 18 double mutants show enhanced resistance during *G. orontii* infection. Recent studies in several different experimental systems have similarly reported that many WRKY transcription families changed their expression levels after powdery mildew infection [53]. These findings indicated that WRKY7, WRKY40, and WRKY41 may play an important role in PM resistance provoked by M7SB41.

### 4.3. M7SB41 Primes SA-Dependent Pathway

Plant hormones SA and JA have been confirmed to form an orderly regulation network for plant-pathogen–endophyte interaction [54]. In order to unravel the SA/JA signaling network governing the protective effect of M7SB41 throughout the PM infection process, the production of SA and JA in tobacco at different PM stages was monitored by pot experiment. It was found that PM infection led to significant SA production in tobacco. Interestingly, this response in E+ plants was faster and stronger than that in E− plants. A large amount of SA accumulated in E+ plants compared with E− plants before and after PM pathogen infection. The concentration of SA in E+ plants reached the highest level during the later stages of PM infection (Figure 2a), while the concentration of JA between E+ and E− plants showed no significant difference throughout the entire infection process (Figure 2b). Similarly, Nomura found that SA accumulation was transiently induced by bacterial PAMP flagellin peptide (flg22) treatment within 6 h in *Arabidopsis*, whereas JA exhibited no significant change in levels [55]. The result suggest that SA has vital roles in PTI. In fact, both PTI and ETI can induce SA accumulation [56]. These findings support the notion that M7SB41 triggers PTI and ETI, which probably contribute to the accumulation of SA levels.

Further, RNA-Seq analysis indicated that TGA1-like, TGA-1a-like, TGA6 up-regulated at 0 h and 24 h, while TGA6-like down-regulated at 72 h in E+ plants (Table S4). The essential role of several transcriptional regulator TGA genes in the SA-dependent pathway has been well characterized [57]. TGA2, TGA3, TGA5, and TGA6 can recruit the transcriptional coactivator NPR1 to the promoters of SA-responsive genes [58]. In contrast, clade I TGA (TGA1, TGA4) act through NPR1-independent pathways [58]. They are required for resistance against virulent pathogens. They effectively regulate the biosynthesis of SA and pipecolic acid (Pip) [59]. These results suggested that tobacco TGA family may be involved in SA-related defense activated by M7SB41. However, JA-marker genes (PI II and MC) and other key JA-related defense genes such as JAZ, MYC2, COI1 were not observed in differential expression between E+ and E− plants (Table S4). Supporting our RNA-seq data, these findings are consistent with previous finding that JA content showed no significant difference between E+ and E− plants. It seems that M7SB41 does not prime JA-related gene expression.

Moreover, we also analyzed the expression of known marker genes for SA-mediated defense signaling by RNA-seq. The results showed that *PR-1* like, *PR-5* like, *PR-5* like isoform, and *PR-10a* like were induced in E+ plants following inoculation with *Seimatosporium* sp. (Table S4). *PR-1* like genes were up-regulated at 0 h and down-regulated at 72 h in E+ plants. *PR1* genes are widely known as prime marker genes in SA-signaling [60]. In tobacco, it has been well established that *PR1* proteins are crucial for resistance against pathogens [61]. *PR-1* genes were described to be induced by both biotrophic and necrotrophic pathogens [60]. In addition to *PR1*, *PR5* also associated with SA-mediated defense pathway [62]. The expression levels of *PR-5* like and/or *PR-5* like isoform were also much higher at 0 h and 24 h in E+ plants. This is consistent with the aforementioned that overexpression of WRKY41 is involved in the regulation of expression of *PR5*. In addition, *PR10* may contribute to an enhanced disease resistance to phytopathogenic fungi. Agarwal et al. [63] reported that overexpression of *JcPR-10a* (*PR-10a* gene from *Jatropha curcas*) in tobacco leads to protection against *Macrophomina phaseolina* fungus.

Accordingly, our data support the notion that, in contrast to priming of JA signaling, SA signaling plays a major role in M7SB41-induced protection during PM infection.

### 4.4. Activation of Phenylpropanoid Biosynthesis Pathway by M7SB41

Phytopathogenic fungi. attack the cell wall is the first stage that triggers the phenylpropanoid biosynthesis pathway for plant defense responses, including deposition of different calloses, production of flavonoids, phenolic acids, and phytoalexins, lignifications, and defense papillae formation [64]. In this work, some key players in the phenylpropanoid pathway, such as PAL, 4CL, HCT, HCT-like were up-regulated in E+ plants (Table S4). Other POD and POD-like genes were also induced by M7SB41 in the whole process of PM infection (Table S4). Similarly, Kurth et al. [65] found that PAL and POD activated by plant-beneficial bacteria *Streptomyces* spp. may enhance oak PM resistance. PAL functions as the first enzyme of the phenylpropanoid pathway and catalyzes the conversion of phenylalanine to trans-cinnamic acid [66]. As anotherroute, it is believed that one of the ways of SA biosynthesis is through PAL catalyzed steps [67]. Thus, PAL, which might be involved in M7SB41-mediated resistance against PM, is linked to positively regulate SA biosynthesis. Furthermore, activation of the phenylpropanoid pathway is generally related with the production of flavonoids (quercetin, kaempferol, naringenin), coumarins, and lignins. These products subsequently represent potential phytoalexins, phytoanticipins, anthocyanins, and other antimicrobial substances important in plant defense. Lignins are involved in plant disease defense through increasing plant cell wall stability.

### 4.5. Different Responses to PM Stress

DEG sets at the same time points between E+ and E− plants were analyzed (Figure 3). At 0 h, significant amounts of DEGs were expressed, suggesting there were significant differences between E+ and E− before PM infection. Promoting endogenous defense responses by M7SB41 might also play a defensive role against PM, during the early stages of *G. cichoracearum* infection. Moreover, dynamic gene expression patterns of E+ and E− plants in key transitional states were analyzed. Eight models of distinct and notable dynamic expression patterns were obtained. Interestingly, it was found that there was a delay in transcriptional responses to PM pathogen stress in E+ plants (72 h) compared with E−plants (24 h) (Figure 4). One possibility for this phenomenon may be that endophyte M7SB41 might be recognized as an alien organism by tobacco and needs to confront the tobacco defense system to colonize. Meanwhile, the activation of immune response is costly, which might cause slow response. Such molecular changes eventually affect later alterations in plant physiology and pathology.

**Figure 7:**
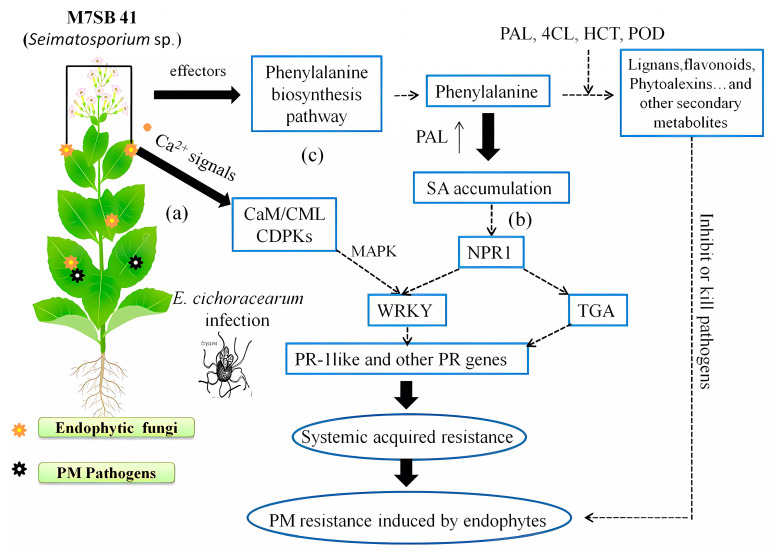
Model for priming of SA-dependent pathway and other defense responses by endophyte M7SB41 against *G. cichoracearum* invasion. Colonization with M7SB41 was associated with activation of Ca^2+^-signaling, SA-signaling, and phenylpropanoid pathway. (**a**) Ca^2+^-signaling: after infection by M7SB41, it may trigger an influx of calcium ions which is then followed by changes in the expression of CaM/CML, CDPKs genes. After that, the expression of downstream WRKY may be regulated. (**b**) SA-signaling: pathogens attack results in increased biosynthesis of SA via PAL pathway. SA accumulation disrupts the oligomeric NPR1 into its monomers and then NPR1 monomers interact with TGA and WRKY. Subsequently, it may induce expression of disease resistance genes including PR genes. Finally, SAR was activated. (**c**) Phenylpropanoid pathway.

In conclusion, comparative transcriptome analysis revealed that Ca^2+^ signaling, SA signaling, and phenylpropanoid biosynthesis pathways of host plants were induced by endophyte M7SB41, and these vital pathways triggered a sufficient defense response to fend off invading PM pathogens (Figure 7). Remarkably, both pot experiments and transcriptomes indicated that, in contrast to priming of JA-dependent defenses, regulation of the SA-dependent defenses seems to play a pivotal role in M7SB41 systemic protection during *G. cichoracearum* infection. In addition, we also confirmed the capacity of M7SB41 to produce cell wall degrading enzymes, β-1, 3-glucanase. Other interesting findings included the elevated expression of *PR1* and *PR5* genes at 0 h. A number of defense-related genes and key enzymes involved in the recognition and signaling of PM resistance were identified, thus providing important clues for better understanding of the mechanisms of endophyte-mediated host plants resistance against pathogens in biological control. Nevertheless, in the current study, the host plant is the model plant tobacco, not wild *Rosa*. Whether M7SB41 could confer powdery mildew resistance in susceptible roses, awaits to be elucidated in further studies.

## Figures and Tables

**Table 1:** The sequencing results of gene expression profiling library. T and C: Treatment (E+ plants, inoculated with endophytes *Seimatosporium* sp.) and control (E− plants, without *Seimatosporium* sp. inoculation) respectively; 0, 24, and 72 represent 0, 24, and 72 h of infection with powdery mildew, respectively; 1, 2, and 3 represent triplicate samples.

Sample	High-quality Clean Reads	Clean Bases	Q30 (%)	GC (%)
T0-1	37,305,518	5.68 G	95.75	43.93
T0-2	33,944,528	5.17 G	95.67	44.04
T0-3	35,193,784	5.36 G	95.67	44.39
T24-1	36,475,460	5.56 G	95.89	44.40
T24-2	39,366,072	5.99 G	95.92	44.38
T24-3	30,943,532	4.72 G	91.61	44.43
T72-1	27,731,788	4.23 G	91.75	44.78
T72-2	39,708,166	6.03 G	96.16	43.91
T72-3	38,035,928	5.78 G	96.27	44.20
C0-1	42,356,674	6.45 G	95.69	44.32
C0-2	41,670,394	6.34 G	95.80	44.30
C0-3	44,483,962	6.77 G	95.75	44.49
C24-1	38,338,732	5.82 G	96.32	44.32
C24-2	45,737,218	6.94 G	96.40	44.78
C24-3	46,247,910	7.02 G	96.16	44.66
C72-1	48,945,184	7.43 G	96.32	44.62
C72-2	36,685,138	5.58 G	95.91	43.66
C72-3	39,491,558	6.01 G	95.78	43.64
Average	39,036,753	5.94 G	95.49	44.29

## Data Availability

The data that support the findings of this study are available from the corresponding author upon reasonable request.

## Supplementary Materials

The following supporting information can be downloaded at: https://www.mdpi.com/article/10.3390/jof9060620/s1, Figure S1: Chromatogram and mass spectrum of salicylic acid in tobacco leaves of E+ and E− group. (a) and (b) represent the total ion chromatogram, quantitative ion and qualitative ion of salicylic acid in E+ and E− group, respectively; Figure S2: E− vs. E+ DEGs volcano plot. Volcano plot of the differentially expressed genes (E− vs. E+) at (a) 0, (b) 24 and (c) 72 hr of infection with powdery mildew. Red: Significantly up-regulated genes; Green: Significantly down-regulated genes; Black: Not differential expressed; Figure S3 Gene ontology (GO) classification histogram of E− vs. E+ DEG. Gene ontology classification of the differentially expressed genes at (a) 0, (b) 24 and (c) 72 hr of infection with powdery mildew; Red: Significantly up-regulated genes; Green: Significantly down-regulated genes; Figure S4 KEGG enrichment bubble chart of E− vs. E+ DEGs. KEGG enrichment bubble chart of the differentially expressed genes at (a) 0, (b) 24 and (c) 72 hr of infection with powdery mildew. Q value is characterized by different colors, as shown in the example on the right, and red indicates Q < 0.05; Figure S5 KEGG enrichment bubble chart of profile 1 and profile 4. (a) (b) showed KEGG enrichment bubble chart of profile1 in E− and E+ plants, respectively; (c) (d) showed KEGG enrichment bubble chart of profile4 in E− and E+ plants, respectively. Q value is characterized by different colors, as shown in the example on the right; Table S1 UHPLC–MS/MS parameters for the quantification of SA; Table S2 Primers used for qRT-PCR validation; Table S3 The results of mapped reads of 18 samples. T: Treatment (E+ plants, inoculated with endophytes *Seimatosporium* sp.); C: control (E− plants, without *Seimatosporium* sp. inoculation) respectively; 0, 24 and 72 represent 0, 24 and 72 hours of infection with powdery mildew, respectively; 1, 2, and 3 represent triplicate samples; Table S4 Differential gene expression levels of selected up- or downregulated genes. Pairwise comparisons C0 vs. T0, C24 vs. T24, C72 vs. T72 are shown. T: Treatment (E+ plants, inoculated with endophytes *Seimatosporium* sp.); C: control (E− plants, without *Seimatosporium* sp. inoculation) respectively; 0, 24 and 72 represent 0, 24 and 72 hours of infection with powdery mildew, respectively.
