# Supplementary material for: Wild Rosa Endophyte M7SB41-Mediated Host Plant’s Powdery Mildew Resistance
Source: J Fungi (Basel). 2023 May 27;9(6):620. doi: 10.3390/jof9060620 (PMC10301872; doi:10.3390/jof9060620)
Supplement: Supplementary file 1 [file jof-09-00620-s001.zip › jof-2368921-supplementary.pdf]

Supplement Materials

Wild *Rosa* endophyte M7SB41-mediated host plant's powdery mildew resistance

Yi Zhao, Wenqin Mao, Wenting Tang, Marcos Antônio Soares, Haiyan Li*

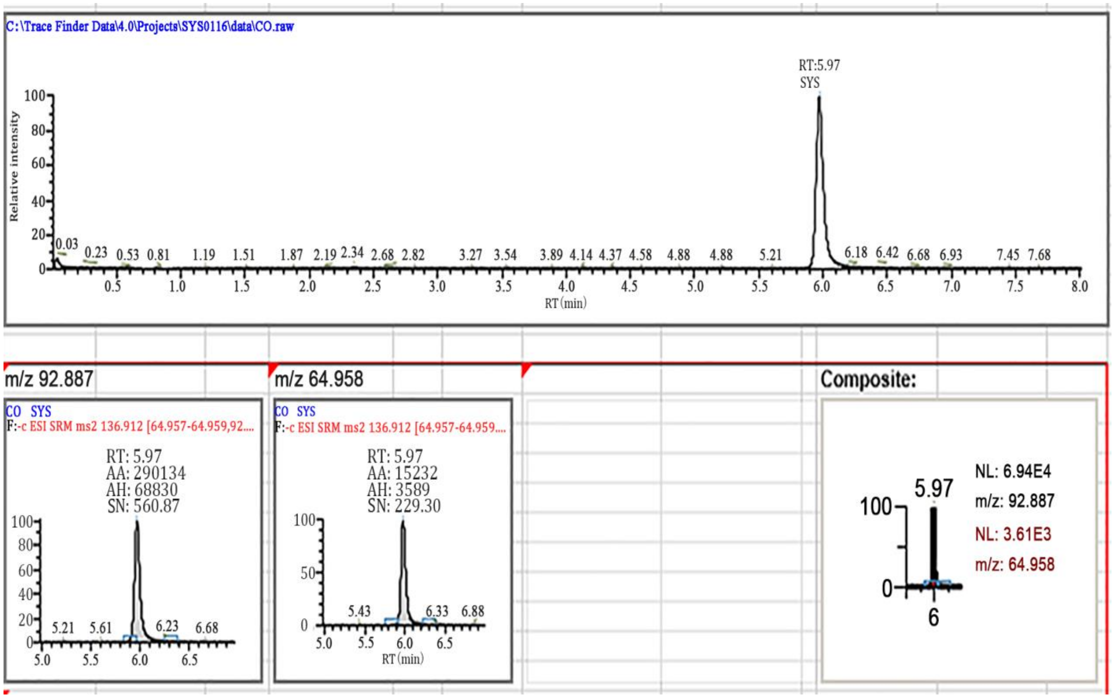

(a)

*: Corresponding author, E-mail: lhyxrn@163.com; Tel. +86 (871)5920751; Fax +86 (871) 5920570.

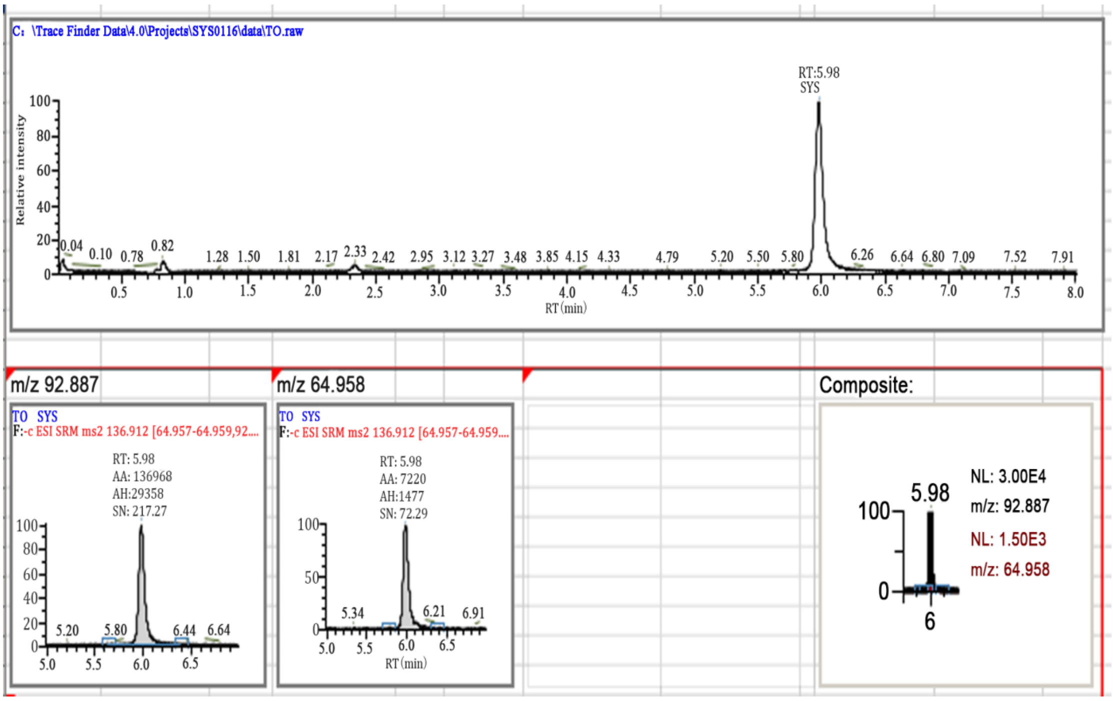

(b)

Fig. S1 Chromatogram and mass spectrum of salicylic acid in tobacco leaves of E+ and E- group. (a) and (b) represent the total ion chromatogram, quantitative ion and qualitative ion of salicylic acid in E + and E - group, respectively.

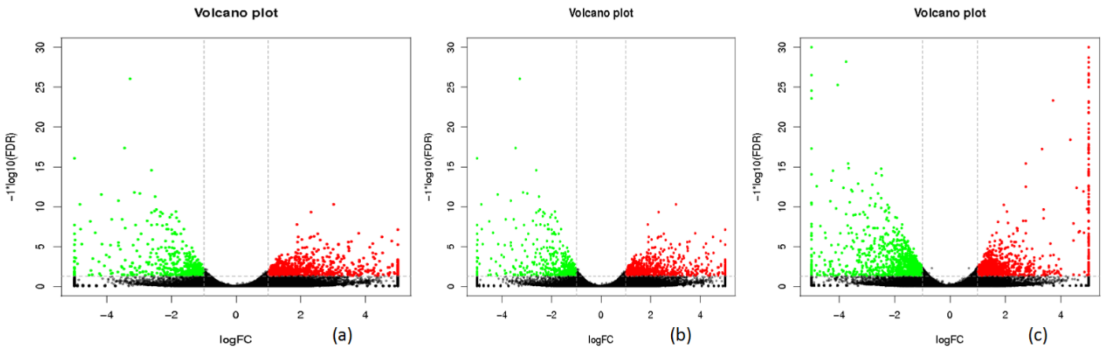

Fig. S2 E- vs E+ DEGs volcano plot. Volcano plot of the differentially expressed genes (E- vs E+) at (a) 0, (b) 24 and (c) 72 hr of infection with powdery mildew. Red: Significantly up-regulated genes; Green: Significantly down-regulated genes; Black: Not differential expressed.

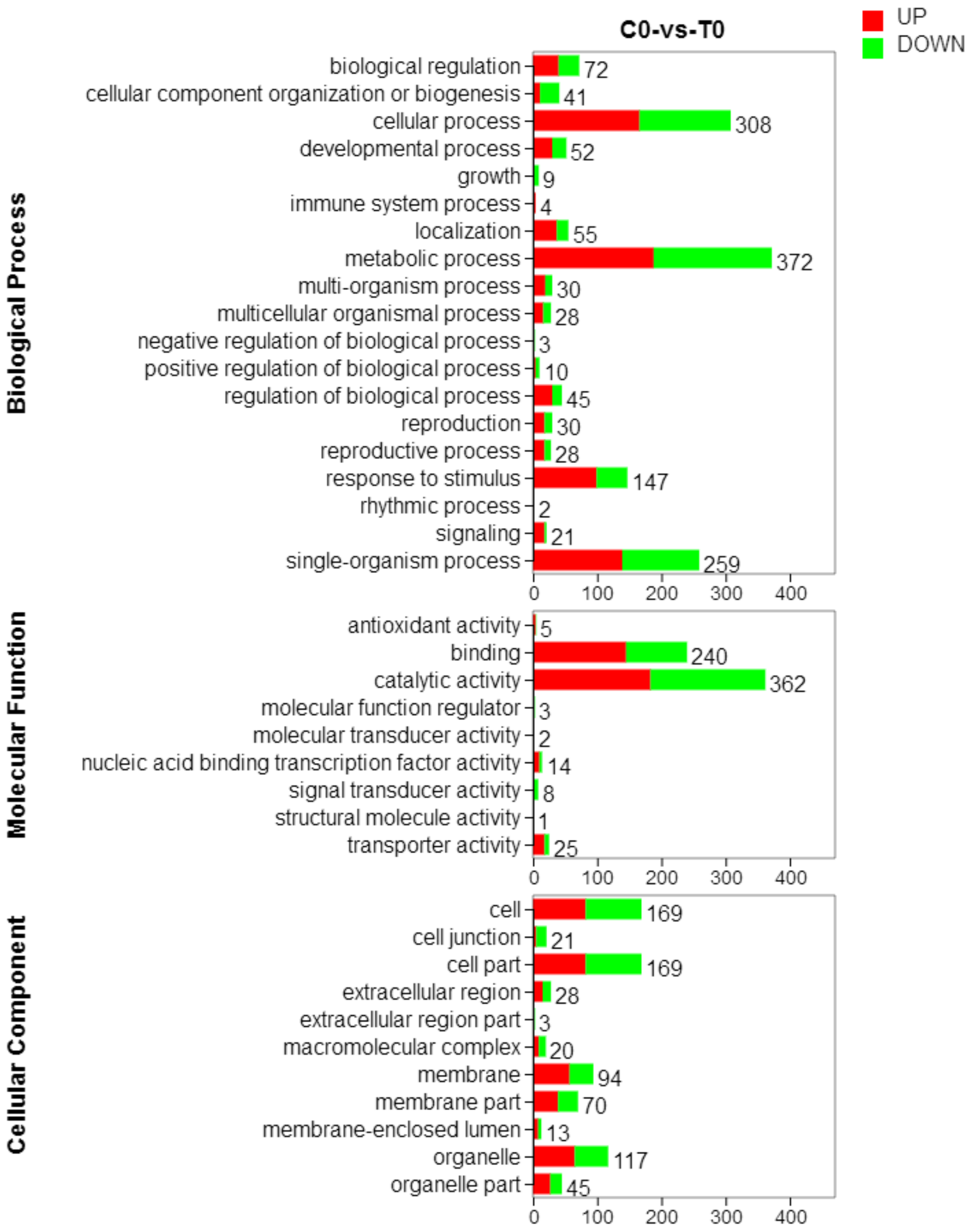
C0-vs-T0
UP
DOWN
Biological Process
biological regulation
cellular component organization or biogenesis
cellular process
developmental process
growth
immune system process
localization
metabolic process
multi-organism process
multicellular organismal process
negative regulation of biological process
positive regulation of biological process
regulation of biological process
reproduction
reproductive process
response to stimulus
rhythmic process
signaling
single-organism process
72
41
308
52
9
4
55
372
30
28
3
10
45
30
28
147
2
21
259
0
100
200
300
400
Molecular Function
antioxidant activity
binding
catalytic activity
molecular function regulator
molecular transducer activity
nucleic acid binding transcription factor activity
signal transducer activity
structural molecule activity
transporter activity
5
240
362
3
2
14
8
1
25
0
100
200
300
400
Cellular Component
cell
cell junction
cell part
extracellular region
extracellular region part
macromolecular complex
membrane
membrane part
membrane-enclosed lumen
organelle
organelle part
169
21
169
28
3
20
94
70
13
117
45
0
100
200
300
400

(a)

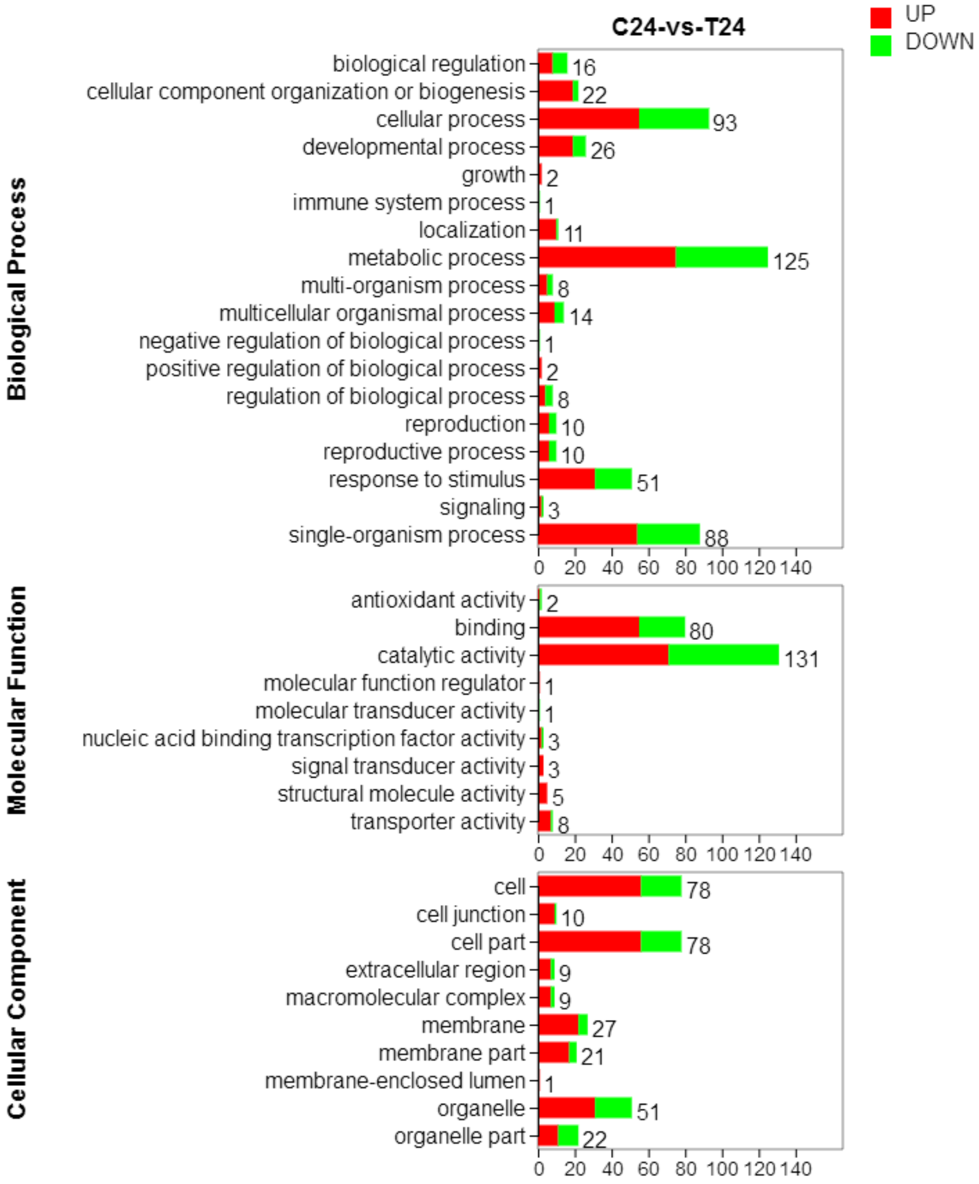
C24-vs-T24
UP
DOWN
Biological Process
biological regulation
16
cellular component organization or biogenesis
22
cellular process
93
developmental process
26
growth
2
immune system process
1
localization
11
metabolic process
125
multi-organism process
8
multicellular organismal process
14
negative regulation of biological process
1
positive regulation of biological process
2
regulation of biological process
8
reproduction
10
reproductive process
10
response to stimulus
51
signaling
3
single-organism process
88
0 20 40 60 80 100 120 140
Molecular Function
antioxidant activity
2
binding
80
catalytic activity
131
molecular function regulator
1
molecular transducer activity
1
nucleic acid binding transcription factor activity
3
signal transducer activity
3
structural molecule activity
5
transporter activity
8
0 20 40 60 80 100 120 140
Cellular Component
cell
78
cell junction
10
cell part
78
extracellular region
9
macromolecular complex
9
membrane
27
membrane part
21
membrane-enclosed lumen
1
organelle
51
organelle part
22
0 20 40 60 80 100 120 140

(b)

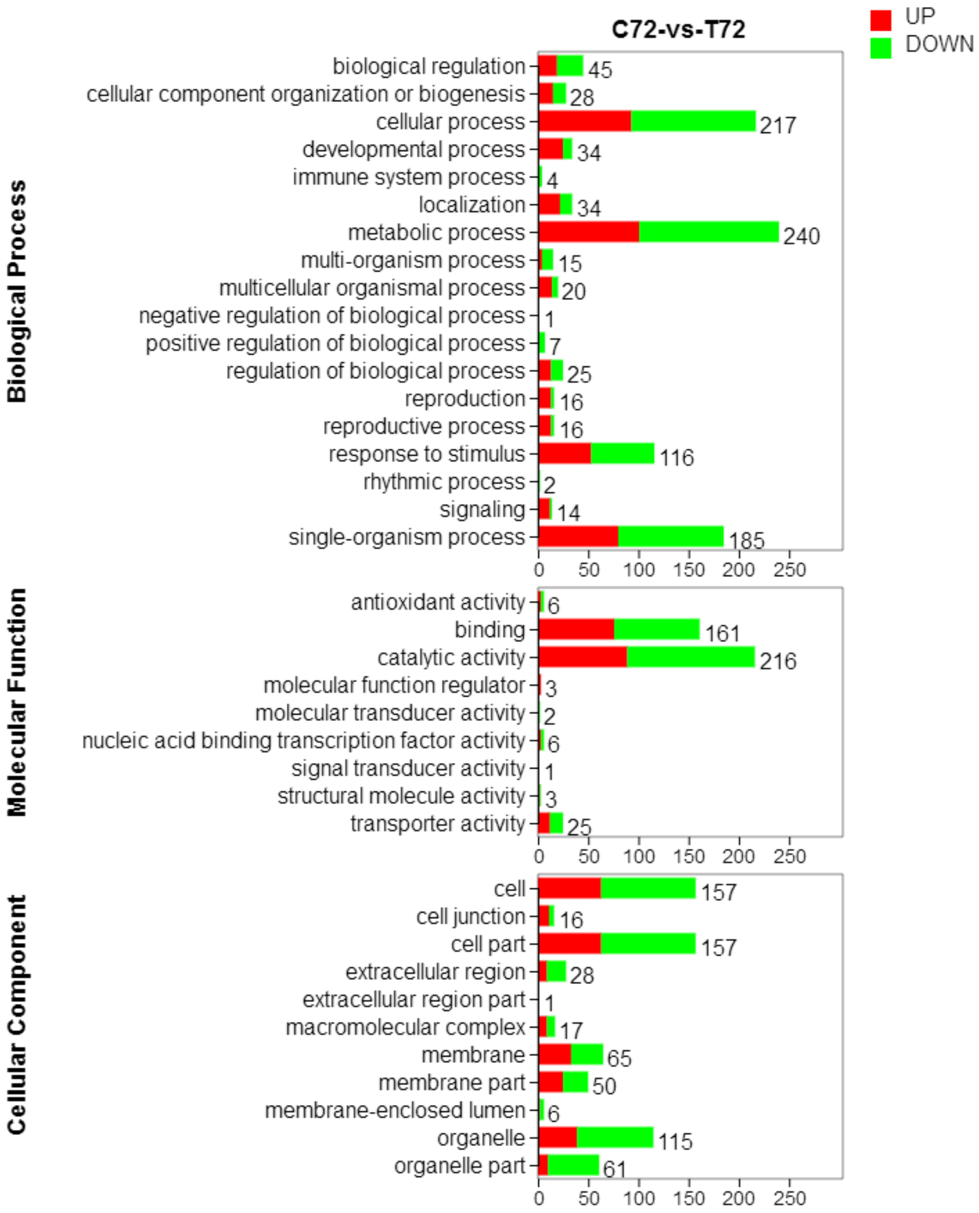

(c)

Fig. S3 Gene ontology (GO) classification histogram of E- vs E+ DEG. Gene ontology classification of the differentially expressed genes at (a) 0, (b) 24 and (c) 72 hr of infection with powdery mildew; Red: Significantly up-regulated genes; Green: Significantly down-regulated genes.

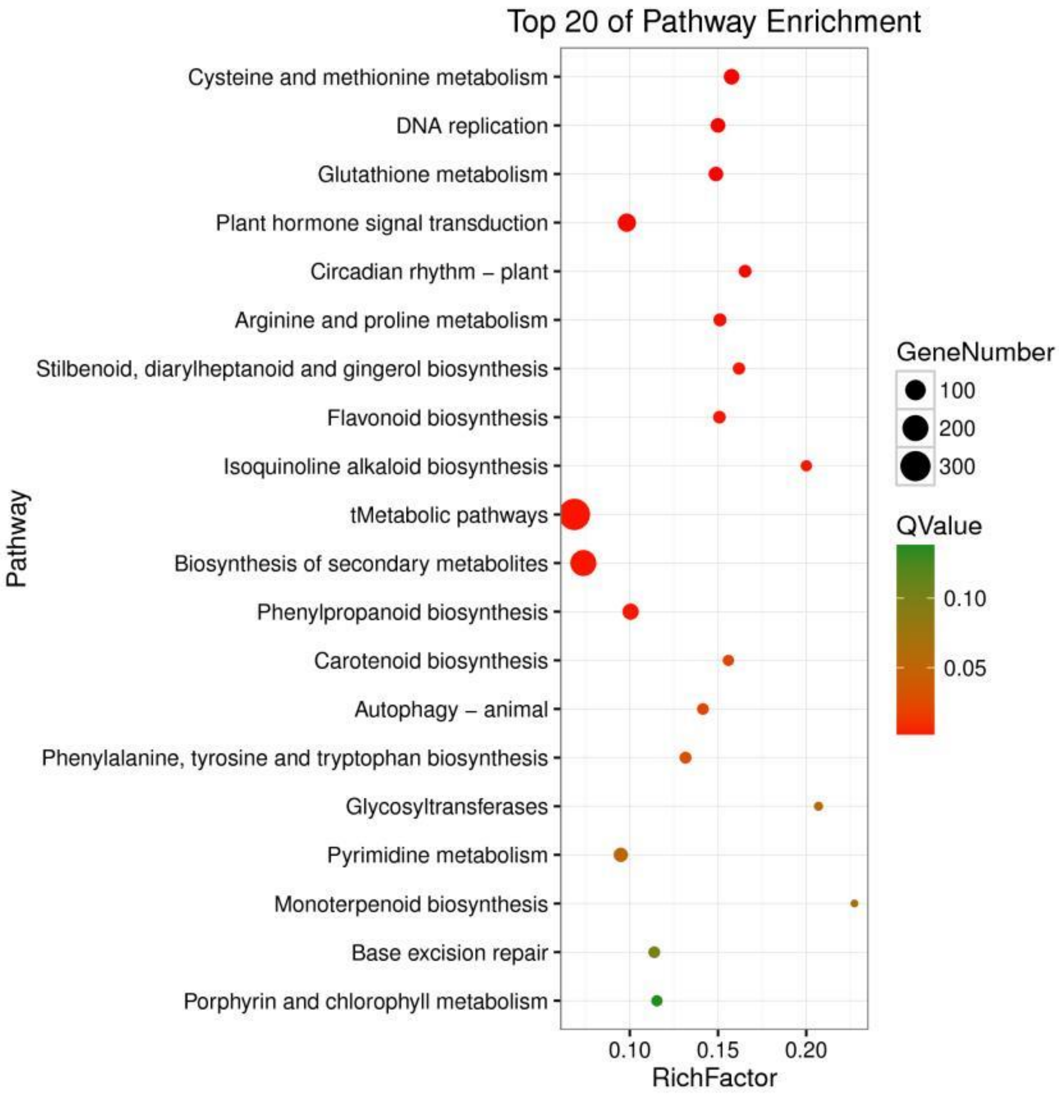

Top 20 of Pathway Enrichment
Cysteine and methionine metabolism
DNA replication
Glutathione metabolism
Plant hormone signal transduction
Circadian rhythm – plant
Arginine and proline metabolism
Stilbenoid, diarylheptanoid and gingerol biosynthesis
Flavonoid biosynthesis
Isoquinoline alkaloid biosynthesis
tMetabolic pathways
Biosynthesis of secondary metabolites
Phenylpropanoid biosynthesis
Carotenoid biosynthesis
Autophagy – animal
Phenylalanine, tyrosine and tryptophan biosynthesis
Glycosyltransferases
Pyrimidine metabolism
Monoterpenoid biosynthesis
Base excision repair
Porphyrin and chlorophyll metabolism
Pathway
0.10
0.15
0.20
RichFactor
GeneNumber
100
200
300
QValue
0.10
0.05

(a)

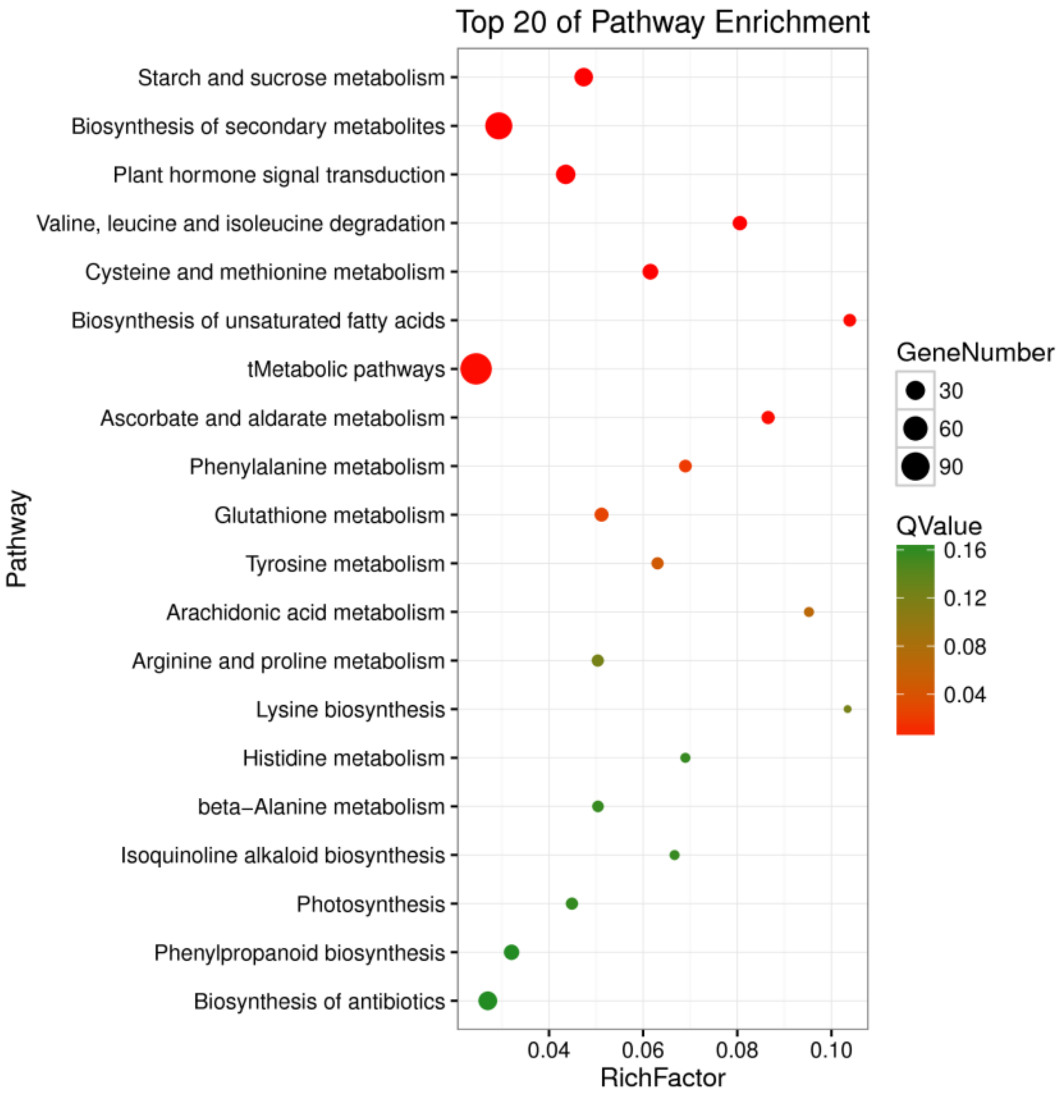

Top 20 of Pathway Enrichment
Starch and sucrose metabolism
Biosynthesis of secondary metabolites
Plant hormone signal transduction
Valine, leucine and isoleucine degradation
Cysteine and methionine metabolism
Biosynthesis of unsaturated fatty acids
tMetabolic pathways
Ascorbate and aldarate metabolism
Phenylalanine metabolism
Glutathione metabolism
Tyrosine metabolism
Arachidonic acid metabolism
Arginine and proline metabolism
Lysine biosynthesis
Histidine metabolism
beta–Alanine metabolism
Isoquinoline alkaloid biosynthesis
Photosynthesis
Phenylpropanoid biosynthesis
Biosynthesis of antibiotics
Pathway
0.04
0.06
0.08
0.10
RichFactor
GeneNumber
30
60
90
QValue
0.16
0.12
0.08
0.04

(b)

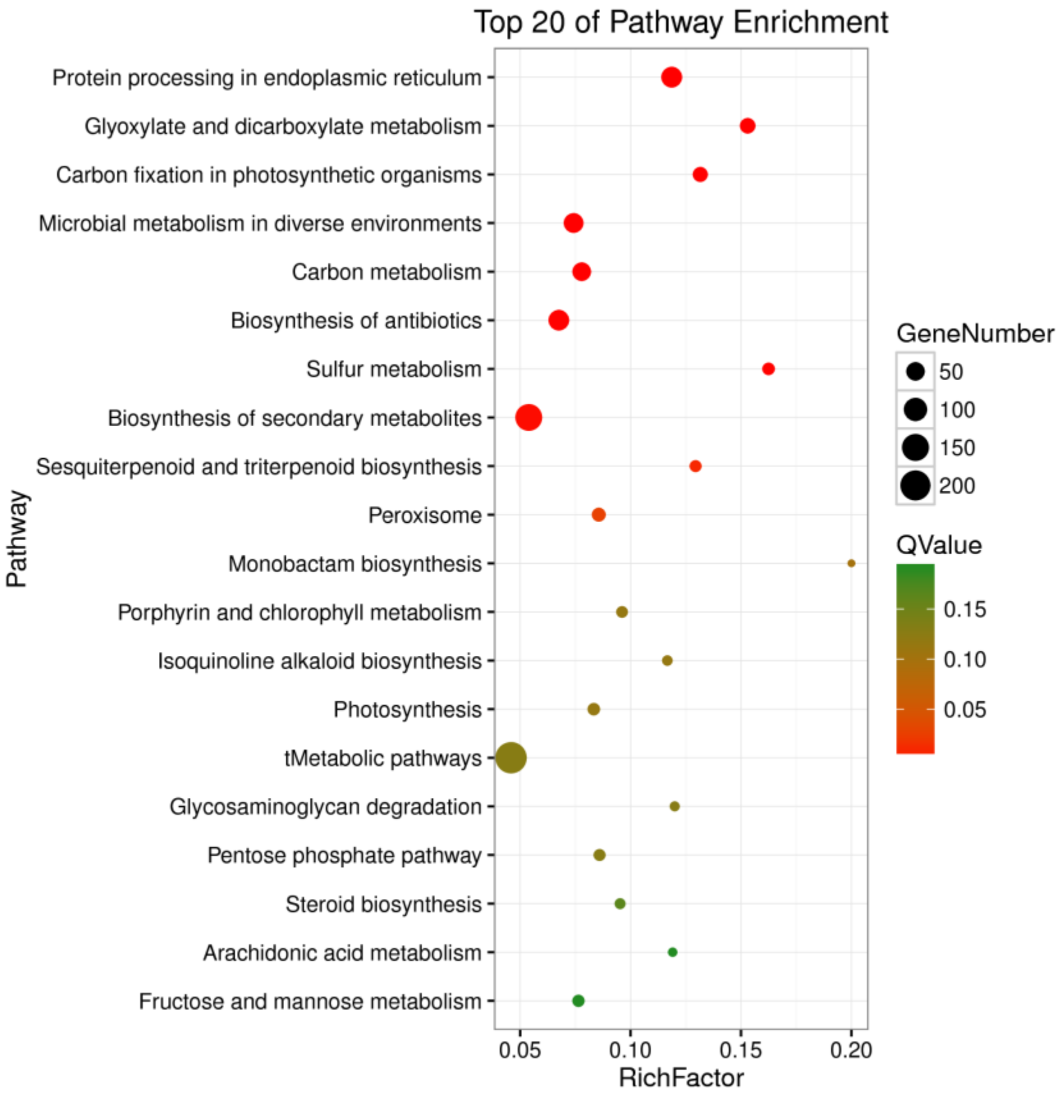

(c)

Fig. S4 KEGG enrichment bubble chart of E- vs E+ DEGs. KEGG enrichment bubble chart of the differentially expressed genes at (a) 0, (b) 24 and (c) 72 hr of infection with powdery mildew. Q value is characterized by different colors, as shown in the example on the right, and red indicates Q< 0.05.

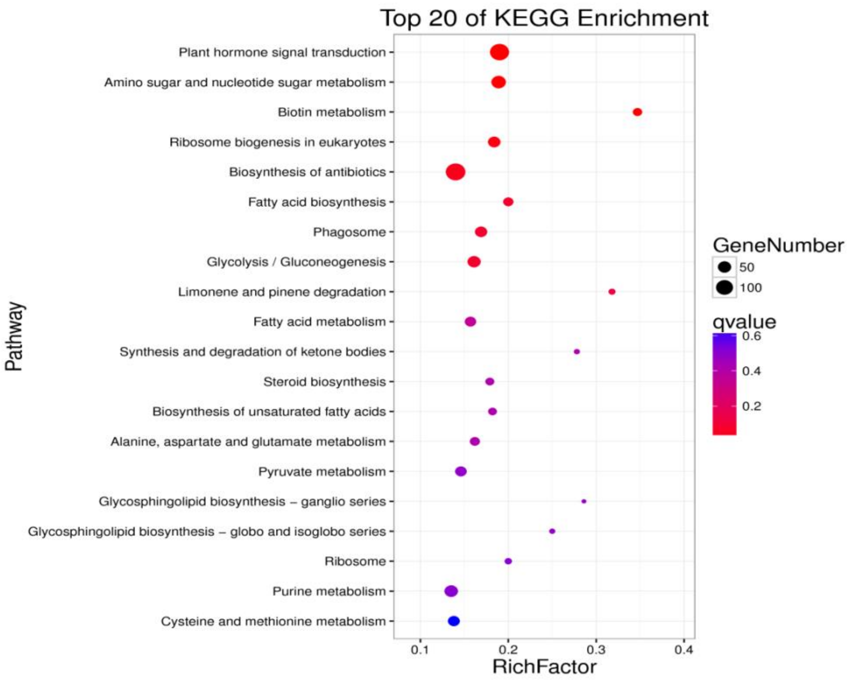
Top 20 of KEGG Enrichment
Plant hormone signal transduction
Amino sugar and nucleotide sugar metabolism
Biotin metabolism
Ribosome biogenesis in eukaryotes
Biosynthesis of antibiotics
Fatty acid biosynthesis
Phagosome
Glycolysis / Gluconeogenesis
Limonene and pinene degradation
Fatty acid metabolism
Synthesis and degradation of ketone bodies
Steroid biosynthesis
Biosynthesis of unsaturated fatty acids
Alanine, aspartate and glutamate metabolism
Pyruvate metabolism
Glycosphingolipid biosynthesis – ganglio series
Glycosphingolipid biosynthesis – globo and isoglobo series
Ribosome
Purine metabolism
Cysteine and methionine metabolism
Pathway
0.1
0.2
0.3
0.4
RichFactor
GeneNumber
50
100
qvalue
0.6
0.4
0.2

(a)

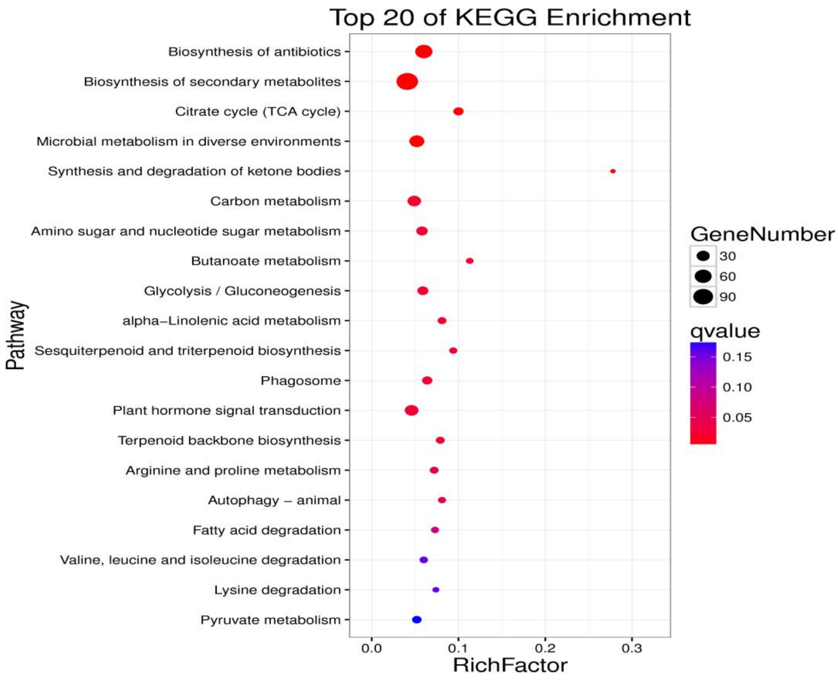
Top 20 of KEGG Enrichment
Biosynthesis of antibiotics
Biosynthesis of secondary metabolites
Citrate cycle (TCA cycle)
Microbial metabolism in diverse environments
Synthesis and degradation of ketone bodies
Carbon metabolism
Amino sugar and nucleotide sugar metabolism
Butanoate metabolism
Glycolysis / Gluconeogenesis
alpha–Linolenic acid metabolism
Sesquiterpenoid and triterpenoid biosynthesis
Phagosome
Plant hormone signal transduction
Terpenoid backbone biosynthesis
Arginine and proline metabolism
Autophagy – animal
Fatty acid degradation
Valine, leucine and isoleucine degradation
Lysine degradation
Pyruvate metabolism
Pathway
0.0
0.1
0.2
0.3
RichFactor
GeneNumber
30
60
90
qvalue
0.15
0.10
0.05

(b)

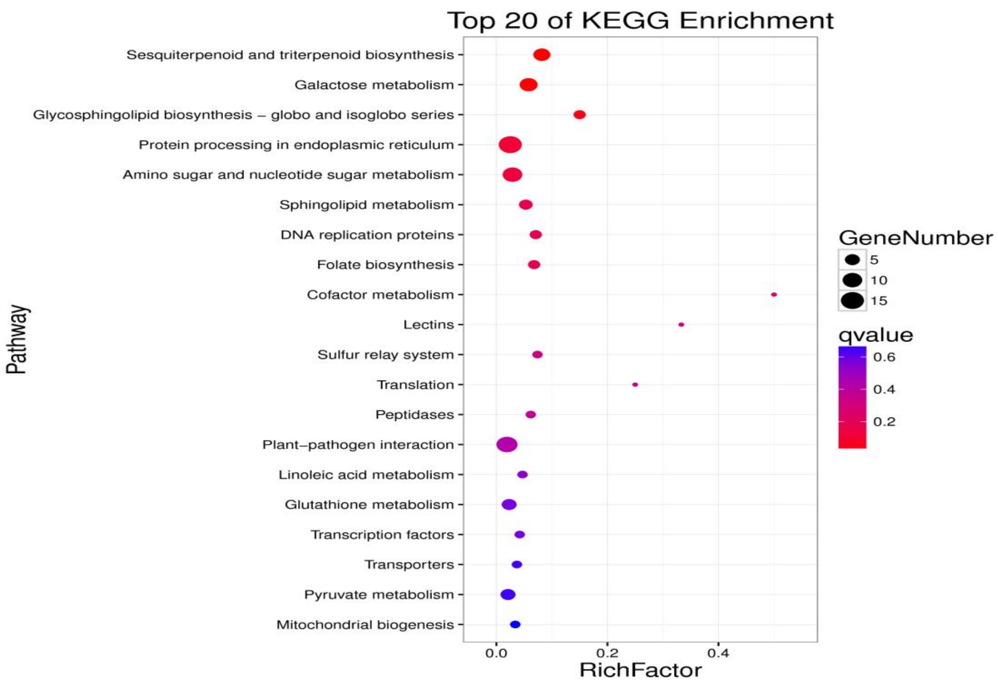

(c)

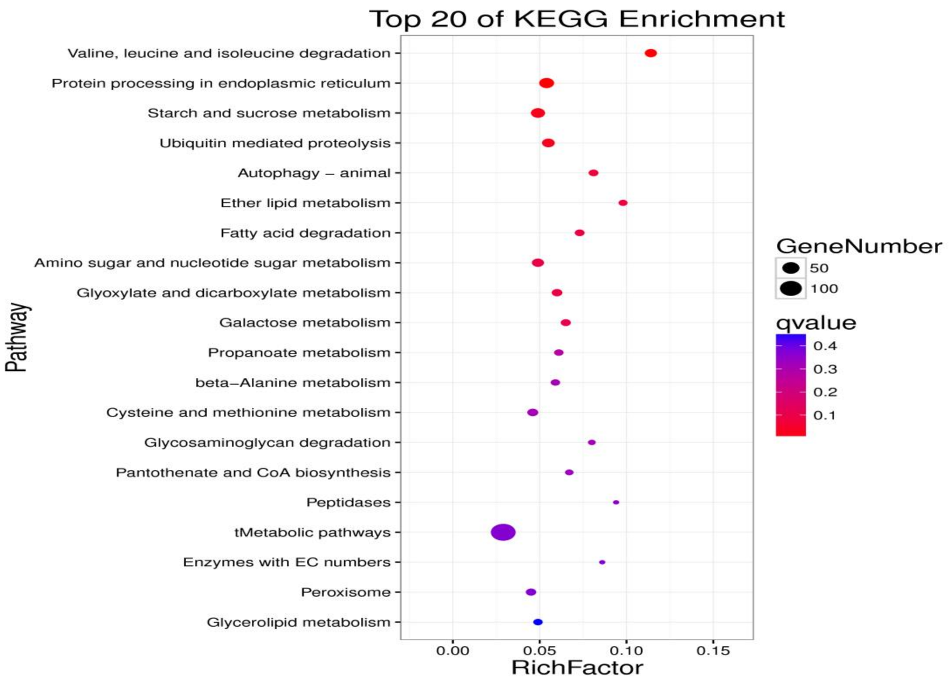

(d)

Fig. S5 KEGG enrichment bubble chart of profile 1 and profile 4. (a) (b) showed KEGG enrichment bubble chart of profile1 in E-and E+ plants, respectively; (c) (d) showed KEGG enrichment bubble chart of profile4 in E- and E+ plants, respectively. Q value is characterized by different colors, as shown in the example on the right.

Table S1 UHPLC–MS/MS parameters for the quantification of SA

Compound	Polarity	Precursor (m/z)	Product (m/z)	Collision energy(v)	RF Lens (v)
Salicylic acid	Negative	136.912	64.958	27.55	57
	Negative	136.912	92.887	15.61	57

Table S2 Primers used for qRT-PCR validation

Gene	Accession number	Primer name	Primer sequence
Ef1α	AF120093	Ef1α-F	GTATGATGAAATCGTGAAGG
		Ef1α-R	CAGAGATGGGGACAAAGG
JAR1-like	XM_016588992.1	JAR1-like isoform-F	GTCACTGTCCCGTCCATA
isoform		JAR1-like isoform-R	CGTTTACTCCAACCCATC
WRKY40	XM_016651389.1	WRKY40-F	GTGAAAGATGAGGCAGGT
		WRKY40-R	GTTGTTGGGATTGGAGAT
JAR1-like	XM_016630684.1	JAR1-like isoform-F	TTGGAAGAAAATGGAGGG
isoform		JAR1-like isoform-R	TGTGGGTGACAATGGGTA
CERK1	XM_016603843.1	CERK1-F	GCTGGCTGGTTTGGTTTA
		CERK1-R	TGATGGAGGTGGTCTTCG
BGLU like	XM_016617986.1	BGLU like-F	TCCTCCCATCTACATCAC
		BGLU like-R	TAAAACCAGCATCCCATT
WRKY31	XM_016651798.1	WRKY31-F	ACTTTACTCCCTTGTTCC
		WRKY31-R	ATGTTGAGGCGTTCTGTC
WRKY7	XM_016618988.1	WRKY7-F	CGGTTCTACGGAAAGTCG
		WRKY7-R	AAGGGGTAACGGAGGCAA
RBOHC	XM_016650414.1	RBOHC-F	CAAAGAGGAGGCGAAAGT
		RBOHC-R	CACACAGGGACCCATGAC

Table S3 The results of mapped reads of 18 samples. T: Treatment (E+ plants , inoculated with endophytes *Seimatosporium* sp.); C: control (E- plants, without *Seimatosporium* sp. inoculation) respectively; 0, 24 and 72 represent 0, 24 and 72 hours of infection with powdery mildew, respectively; 1, 2, and 3 represent triplicate samples.

Sample	Total Reads	Unmapped Reads	Unique Mapped Reads	Multiple Mapped Reads	Mapping Ratio
T0-1	35516458	2752088	32215624(90.71%)	548746 (1.55%)	92.25%
T0-2	32860452	2519239	29810829 (90.72%)	530384 (1.61%)	92.33%
T0-3	33375618	2548923	30279133 (90.72%)	547562 (1.64%)	92.36%
T24-1	32862502	2416784	29963176 (91.18%)	482542 (1.47%)	92.65%
T24-2	37285354	2794928	33888022 (90.89%)	602404 (1.62%)	92.50%
T24-3	30025614	3708748	25843280 (86.07%)	473586 (1.58%)	87.65%
T72-1	24636064	3098761	21217297 (86.12%)	320006 (1.30%)	87.42%
T72-2	38199322	2794860	34841404 (91.21%)	563058 (1.47%)	92.68%
T72-3	36475100	2800971	33075431 (90.68%)	598698 (1.64%)	92.32%
C0-1	40703052	3255680	36740564 (90.26%)	706808 (1.74%)	92.00%
C0-2	40452040	3059232	36682720 (90.68%)	710088 (1.76%)	92.44%
C0-3	42052442	3280451	38028503 (90.43%)	743488 (1.77%)	92.20%
C24-1	36932384	2505963	33805849 (91.53%)	620572 (1.68%)	93.21%
C24-2	42284976	3092015	38490313 (91.03%)	702648 (1.66%)	92.69%
C24-3	44180332	3298097	40124741 (90.82%)	757494 (1.71%)	92.53%
C72-1	46034962	3354260	41888924 (90.99%)	791778 (1.72%)	92.71%
C72-2	36042370	2616973	32868117 (91.19%)	557280 (1.55%)	92.74%
C72-3	39031126	2882987	35528513 (91.03%)	619626 (1.59%)	92.61%

Table S4 Differential gene expression levels of selected up- or downregulated genes. Pairwise comparisons C0 vs T0, C24 vs T24, C72 vs T72 are shown. T: Treatment (E+ plants , inoculated with endophytes *Seimatosporium* sp.); C: control (E- plants, without *Seimatosporium* sp. inoculation) respectively; 0, 24 and 72 represent 0, 24 and 72 hours of infection with powdery mildew, respectively.

Accession number	Putative function	C0 vs T0	C24 vs T24	C72 vs T72
XM_016612404.1	TGA1-like	upregulated	-	-
XM_016654927.1	TGA-1a-like	upregulated	-	-
XM_016640845.1	TGA-1a-like	upregulated	-	-
XM_016625627.1	TGA6	-	upregulated	-
XM_016602112.1	TGA6-like	-	-	downregulated
XP_009768533.1	WRKY4	downregulated	-	-
XP_009587051.1	WRKY6	upregulated	-	-
XM_016580199.1	WRKY41	upregulated	-	-
XM_016581187.1	WRKY31	upregulated	-	-
XM_016581234.1	WRKY3	downregulated	-	-
XM_016607873.1	WRKY68	upregulated	-	-
XM_016610715.1	WRKY7	upregulated	upregulated	-
XM_016614949.1	WRKY49	downregulated	-	-
XM_016618988.1	WRKY7	upregulated	-	-
XM_016619314.1	WRKY7	upregulated	-	-
XM_016621642.1	WRKY40	downregulated	-	-
XM_016624990.1	WRKY41 isoform	upregulated	-	-
XM_016625844.1	WRKY11	upregulated	-	-
XM_016631125.1	WRKY65	upregulated	-	-
XM_016631674.1	WRKY22-like	upregulated	-	-
XM_016634340.1	WRKY44	downregulated	-	-
XM_016638899.1	WRKY41	upregulated	-	-
XM_016641002.1	WRKY53	upregulated	-	-
XM_016643087.1	WRKY7	upregulated	-	-
XM_016651798.1	WRKY31	upregulated	-	-
XM_016651389.1	WRKY40	downregulated	-	-
XM_016582122.1	WRKY70 isoform	-	downregulated	-
XM_016606924.1	WRKY69 isoform	-	downregulated	-
XM_016644013.1	WRKY7	-	upregulated	-
XM_016591510.1	WRKY50	-	-	downregulated
XM_016638899.1	WRKY41	-	-	upregulated
ABY59789.1	MES3	upregulated	-	-
XM_016588992.1	JAR1-like isoform	upregulated	-	-

Accession number	Putative function	C0 vs T0	C24 vs T24	C72 vs T72
XM_016630684.1	JAR1-like isoform	upregulated	upregulated	-
XM_016584785.1	4CL	upregulated	-	-
XP_009759945.1	PAL	-	upregulated	-
XM_016588500.1	PAL	-	-	upregulated
XM_016617986.1	BGLU	downregulated	-	-
XLOC_023187	HCT-like	upregulated	-	-
XLOC_044024	HCT-like	upregulated	-	-
ncbi_107768362	HCT-like	upregulated	-	-
ncbi_107772942	HCT-like	upregulated	-	-
ncbi_107789426	HCT-like	upregulated	-	upregulated
ncbi_107796658	HCT	upregulated	-	upregulated
ncbi_107804864	HCT-like	upregulated	-	downregulated
ncbi_107826939	HCT-like isoform	-	-	upregulated
ncbi_107777955	CAD6	downregulated	-	-
ncbi_107789450	CAD1	upregulated	-	-
ncbi_107790164	CAD6	downregulated	-	-
ncbi_107798963	CAD6	upregulated	-	-
ncbi_107816761	CAD2	downregulated	-	-
ncbi_107767921	CCR2-like	upregulated	-	-
XLOC_025770	POD 51-like	upregulated	-	-
ncbi_107759554	POD 51-like	upregulated	-	-
ncbi_107762756	POD 66-like	downregulated	-	-
ncbi_107765883	POD 63-like	downregulated	-	-
ncbi_107770624	POD 12-like	upregulated	-	-
ncbi_107777661	POD 7-like	upregulated	-	-
ncbi_107782926	POD 7-like	upregulated	-	-
ncbi_107794292	POD 63-like	downregulated	upregulated	-
ncbi_107804632	POD 51-like	upregulated	-	upregulated
ncbi_107808759	POD 12-like	upregulated	-	-
ncbi_107813838	POD 12-like	downregulated	-	-
ncbi_107828946	POD 12-like	downregulated	-	-
ncbi_107794830	POD 21	-	upregulated	-
ncbi_107806425	POD3	-	upregulated	upregulated
ncbi_107807567	POD3-like	-	upregulated	upregulated
ncbi_107786793	POD6	-	upregulated	-
ncbi_107758994	POD19	-	-	downregulated
ncbi_107770624	POD12-like	-	-	upregulated
ncbi_107773042	POD N1-like	-	-	upregulated
ncbi_107794830	POD21	-	-	upregulated
ncbi_107815364	POD48-like	-	-	upregulated

Accession number	Putative function	C0 vs T0	C24 vs T24	C72 vs T72
XLOC_059410	CML21	upregulated	-	-
ncbi_107771773	CML18	upregulated	-	upregulated
ncbi_107813448	CML41	downregulated	-	-
ncbi_107817356	CML44	upregulated	-	-
ncbi_107817357	CML44	upregulated	-	-
ncbi_107819435	CML44	upregulated	upregulated	-
ncbi_107823367	CML41	-	upregulated	-
ncbi_107790990	CML1	downregulated	-	-
ncbi_107792965	CML1	downregulated	-	-
ncbi_107803626	CML1	downregulated	-	-
ncbi_107813304	CML3	upregulated	-	-
ncbi_107792571	CaM	-	upregulated	-
ncbi_107763763	CDPK17-like	downregulated	-	-
ncbi_107767341	CDPK -like isoform X2	downregulated	-	-
ncbi_107807275	calmodulin-like	upregulated	upregulated	-
ncbi_107768378	PR-1like	upregulated	upregulated	-
ncbi_107798618	PR-1like	upregulated	-	-
ncbi_107763263	PR-1c	-	-	downregulated
ncbi_107807832	PR-1b	-	-	downregulated
ncbi_107765038	PR-5 like	downregulated	-	-
ncbi_107770507	PR-10a like	upregulated	-	-
ncbi_107830187	PR-10a like	upregulated	-	downregulated
ncbi_107831906	PR-5 like isoform	upregulated	-	downregulated
ncbi_107832140	PR-5 like isoform	upregulated	upregulated	-
ncbi_107804128	PR-5 like	-	upregulated	-
ncbi_107766311	PR-4b	-	-	downregulated
ncbi_107791665	PR-1 like	-	-	downregulated
ncbi_107802320	PR-4a	-	-	downregulated
ncbi_107808479	PR-10a like	-	-	downregulated
ncbi_107823075	PR-10a like	-	-	downregulated
ncbi_107823159	PR-1 like	-	-	downregulated
